# The four weeks before lockdown during the COVID-19 pandemic in Germany: a weekly serial cross-sectional survey on risk perceptions, knowledge, public trust and behaviour, 3 to 25 March 2020

**DOI:** 10.2807/1560-7917.ES.2021.26.42.2001900

**Published:** 2021-10-21

**Authors:** Cornelia Betsch, Lars Korn, Tanja Burgard, Wolfgang Gaissmaier, Lisa Felgendreff, Sarah Eitze, Philipp Sprengholz, Robert Böhm, Volker Stollorz, Michael Ramharter, Nikolai Promies, Freia De Bock, Philipp Schmid, Britta Renner, Lothar H Wieler, Michael Bosnjak

**Affiliations:** 1 University of Erfurt, Erfurt, Germany; 2 Department of Implementation Science, Bernhard Nocht Institute for Tropical Medicine, Hamburg, Germany; 3 Leibniz Institute for Psychology Information and Documentation, Trier, Germany; 4 University of Konstanz, Konstanz, Germany; 5 University of Copenhagen, Copenhagen, Denmark; 6 Faculty of Psychology, University of Vienna, Vienna, Austria; 7 Science Media Center Germany gGmbH, Köln, Germany; 8 Department of Tropical Medicine, Bernhard Nocht Institute for Tropical Medicine and I. Dep. of Medicine University Medical Center, Hamburg-Eppendorf, Germany; 9 Karlsruhe Institute of Technology, Karlsruhe, Germany; 10 Federal Centre for Health Education, Köln, Germany; 11 Robert Koch Institute, Berlin, Germany; 12 University of Trier, Trier, Germany

**Keywords:** coronavirus, COVID-19, policy acceptance, lockdown, knowledge-behaviour gap, cluster analysis, behavioural insights

## Abstract

**Background:**

During the COVID-19 pandemic, public perceptions and behaviours have had to adapt rapidly to new risk scenarios and radical behavioural restrictions.

**Aim:**

To identify major drivers of acceptance of protective behaviours during the 4-week transition from virtually no COVID-19 cases to the nationwide lockdown in Germany (3–25 March 2020).

**Methods:**

A serial cross-sectional online survey was administered weekly to ca 1,000 unique individuals for four data collection rounds in March 2020 using non-probability quota samples, representative of the German adult population between 18 and 74 years in terms of age × sex and federal state (n = 3,910). Acceptance of restrictions was regressed on sociodemographic variables, time and psychological variables, e.g. trust, risk perceptions, self-efficacy. Extraction of homogenous clusters was based on knowledge and behaviour.

**Results:**

Acceptance of restrictive policies increased with participants’ age and employment in the healthcare sector; cognitive and particularly affective risk perceptions were further significant predictors. Acceptance increased over time, as trust in institutions became more relevant and trust in media became less relevant. The cluster analysis further indicated that having a higher education increased the gap between knowledge and behaviour. Trust in institutions was related to conversion of knowledge into action.

**Conclusion:**

Identifying relevant principles that increase acceptance will remain crucial to the development of strategies that help adjust behaviour to control the pandemic, possibly for years to come. Based on our findings, we provide operational recommendations for health authorities regarding data collection, health communication and outreach.

## Introduction

Even before the coronavirus disease (COVID-19) outbreak was officially declared a pandemic in March 2020, several countries had experienced high numbers of cases and deaths, necessitating the introduction of strict measures to prevent physical contact in an effort to slow transmission of the severe acute respiratory syndrome coronavirus 2 (SARS-CoV-2) [1,2]. Non-pharmaceutical interventions were implemented in the absence of specific treatments or preventive vaccines. Responsible authorities had to enact measures and rely on citizens’ trust and compliance, while only able to provide uncertain information, e.g. about the likelihood of contracting COVID-19 or its potential severity [3]. The public was forced to rapidly adapt opinions and perceptions to new risk scenarios and radical behavioural changes. Thus, this early COVID-19 outbreak period is of particular interest from a public health perspective, as it may serve as an exemplary case for the ability of the public to adapt to an emerging infectious disease that is rapidly spreading worldwide.

Many studies were conducted during that time to assess individuals’ risk perceptions, behaviours and trust in information sources, e.g. in the United States [3-8], China [9-11], Vietnam [12], Bangladesh [13], Indonesia [14], South Africa [15], Nigeria [16], Sierra Leone [17], Australia [18], Italy [19,20], Israel [21], Spain [22], Egypt [23] and Japan [24], as well as cross-national comparisons [25-27]. Most analyses relied on cross-sectional, single data collections that were not always representative of the respective country. The results were used to support crisis communication, prepare key messages [28] and facilitate implementation of rational, adaptive and protective behaviour [29]. A few studies used a longitudinal design with two data collections [30-32]; however, they used convenience and snowball sampling, which can increase self-selection bias and does not allow extrapolation to populations. From these studies, we can conclude that risk perceptions changed considerably over time. Thus, building pandemic response strategies based on a single time period may miss prominent changes and trajectories. However, knowledge remains limited about how risk perceptions and acceptance of measures developed from the outset of the pandemic, given the complex and dynamic interplay among changing epidemiology, news media attention and pandemic control measures [33].

Through the COVID-19 Snapshot Monitoring (COSMO) [34,35], weekly data on public opinion and perceptions have been collected since 3 March 2020, and have provided continuous evidence to Germany’s government and crisis managers, as well as health communicators and news media. The distributions of age, sex and federal state in each sample of this serial cross-sectional survey matched the German population for each respective variable. The protocol attracted substantial interest as identified by download statistics at psycharchives.org and built the basis of a study protocol suggested by the World Health Organization (WHO) in July 2020 [36,37]. However, to our knowledge, this study is the only one of its kind that has assessed data from a country that was relatively unaffected at the outset of the study period until the lockdown was imposed, affecting large parts of society and economy. Here, we present data from Germany during this crucial period (3–25 March 2020), which investigates the interplay between disease dynamics, political measures, news media, resulting knowledge and public opinion. These analyses can serve as a blueprint for other studies using a similar protocol and provide recommendations on implementing such survey systems and on how to learn from the data.

Extant research on public health and social measures indicates that topic knowledge, trust in the government and risk perceptions are relevant to explain the acceptance and uptake of such measures [38-40]. This is supported by ample theoretical approaches to understanding preventive behaviour that point in similar directions [41,42], e.g. the Confidence and Cooperation model [43], the protection motivation theory [44,45], the Health Belief Model [46] and the health action process approach [44]. Therefore, our main objective was to identify changes in risk perceptions and behaviours over time and answer the following research questions: (i) How did people perceive the disease and the pandemic situation from the outset of the pandemic? (ii) What did people know about COVID-19 and how quickly were they acquiring knowledge? (iii) How much did they trust institutions and media? (iv) How well did they accept imposed safety measures, and which factors affected acceptance?

Since translating knowledge into preventive health behaviour is a major challenge in health promotion [47], we also assessed which groups were compliant and which had difficulties following safety measures. Such knowledge from this early stage of the pandemic can help steer policies, public health measures and communication strategies in future waves or local outbreaks by identifying relevant principles that should be considered. With such a dynamic situation, fast and effective action may prevent larger outbreaks, whereas ineffective strategies may cause irreversible damage, e.g. avoidable deaths, misinformation or complacency. Thus, identifying factors that facilitate acceptance and compliance early on is crucial [48].

## Methods

### Study context

During the study period (3–25 March 2020), confirmed COVID-19 cases in Germany rose from 145 to 39,441 [49], and the situation transitioned from an issue threatening only other countries, such as China or Italy, into a severe and real threat in Germany. Pandemic control measures shifted drastically from none at all to a strict contact ban, wherein citizens were permitted to meet only one other person, at a distance of at least 1.5 m. The first COVID-19-associated deaths occurred in Germany on 8 March, just before the second week of data collection. The government reacted by closing non-essential shops and schools on 13 March, the day before the third round of data collection. A strict contact ban was enacted on 22 March, 2 days before the fourth round of data collection (Supplementary Figure S1: Timeline of relevant events).

### Study design

This study was designed as a serial, cross-sectional online survey with ca 1,000 unique individuals participating in each of the four rounds of data collection. Care was taken that participants did not participate more than once. Data were collected on 3–4 (round 1), 10–11 (round 2), 17–18 (round 3) and 24–25 March 2020 (round 4).

### Participants

Non-probability quota samples were used, matching the German adult population in terms of age × sex and federal state, according to the German census. Study participants were members of an ISO 26362:2009-compliant online panel [50]. The externally contracted company responsible for data collection financially compensated participants. The questionnaire was programmed by the COSMO research group. All individuals between the ages of 18 and 74 years who completed the survey were eligible for inclusion in the analyses. Participants were admitted to the survey or excluded based on quotas. Response rates, defined as the number of people who participated in relation to the number of people invited, ranged between 15.1% (third data collection round) and 20.2% (second data collection round). We included a total of 3,910 individuals, with n_round 1_ = 973, n_round 2_ = 966, n_round 3_ = 1,015 and n_round 4_ = 956 for each of the four collection rounds. During the third and fourth data collection rounds, one participant was excluded because of previous participation.

### Measures and procedure

At the outset of each of the four data collection rounds, participants received a link to the online questionnaire, which took between 15 and 25 min to complete. During each data collection round, the same core questions assessing demographics and psychological variables were included in the survey. Each questionnaire contained unique questions not reported here. The original questionnaires are accessible online [35].

### Demographic variables

In the survey, participants provided sociodemographic information on their age, sex, education (low: up to 9 years of schooling; medium: at least 10 years of schooling without university entrance qualification; high: at least 10 years of schooling with university entrance qualification), whether or not they worked in the healthcare sector and the size of their community (small: < 20,000 inhabitants; medium: 20,000–100,000 inhabitants; large: > 100,000 inhabitants).

### Psychological variables

All variables are described in detail in the Supplementary Methods [51]. The questionnaire comprised relevant theoretical concepts, as detailed above, to (i) assess trust in governmental and health institutions and media (one item each, using ratings on Likert-type scales ranging from 1 to 7); (ii) knowledge about SARS-CoV-2 and COVID-19 (three right/wrong items); (iii) knowledge about several protective behaviours (summary score); (iv) cognitive risk perception, e.g. susceptibility to SARS-CoV-2 infection (ratings on Likert-type scales ranging from 1 to 7 [52]); (v) affective risk perception, e.g. feeling fear, worry or thinking about the coronavirus all the time [53] (mean score across three Likert-type scales ranging from 1 to 7); (vi) self-efficacy in protecting oneself against the disease (one item, Likert-type scale ranging from 1 to 7); (vii) perceiving the outbreak as media hype (one item; Likert-type scale ranging from 1 to 7); (viii) protective behaviours practised (yes/no) and (ix) acceptance of restrictions of freedom, as a proxy for acceptance of safety measures (one Likert-type scale ranging from 1 to 7).

### Time

We included time as a between-subjects variable to assess whether the dependent variables changed over time.

### Google Trends search volume

Google Trends data indicate how often certain keywords are searched on the internet and serve as a proxy for information demand and attention paid to a topic. Data on the Google search volume were downloaded from Google Trends [54]. In contrast to common words used in the scientific literature, news outlets and laypeople in Germany used the term ‘corona’ much more frequently than COVID-19 or SARS-CoV-2; therefore, we focussed on the search term ‘corona’ only. Thus, the data represent only the search interest regarding ‘corona’, relative to the maximum interest (number of searches) for Germany between 25 February and 25 March 2020. As provided by Google Trends, a value of 100 indicated peak popularity for the term, 50 indicated moderate popularity and zero indicated that there was not enough data for this term.

### Newspaper volume data

WHO noted that, along with the COVID-19 pandemic, there has also been an ‘infodemic’, i.e. an abundance of information that includes misinformation [55]. Considering that news media are an important source of health information [56], we used the frequency with which news outlets reported about the COVID-19 pandemic as an indicator of information density and availability. To study the amount of newspaper reporting in Germany during the study period and the week prior (25 February–25 March 2020), we collected data from the Wiso press database (https://www.wiso-net.de) using the filter ‘Presse Deutschland’ to exclude articles from Switzerland and Austria. Altogether, 158 newspapers, magazines and online news portals generated hits within the search strings for all texts containing the keywords ‘corona’ or ‘covid’.

### Power and precision

We chose a large sample size to detect small effects and to increase the probability of congruence between the distribution of the demographics in the sample and the German population (regarding age × sex and federal state), comprising approximately 1,000 individuals per data collection round [57]. Deviations occurred because of decreased or increased invitations to participants in the face of open quotas. Given a sensitivity power analysis for zero-order correlations (p = 0.05), a sample size of 1,000 is sufficient to detect correlation coefficients of (at least) r = 0.08 with sufficient power of 0.8 in each survey.

### Statistical analysis

We used unweighted [58], stepwise linear regression analyses with backward elimination as follows: (i) first step: demographic variables and time, (ii) second step: psychological variables and (iii) third step: interactions between psychological variables and time. Statistical requirements were tested and fulfilled (see the Supplement). To identify which variables affected acceptance of restrictive measures, we used ordinary least squares regressions (packages lm, glm) in the statistical software R (R Foundation, Vienna, Austria) plus the modern applied statistics with S (MASS) package [59]. We further examined acceptance of new behavioural rules over time and factors influencing this acceptance by using a classification-type approach to extract profiles from homogenous groups of those who complied or did not comply with the requested behaviour. For extraction of homogenous groups, we used a k-means cluster analysis and multinomial logistic regression to examine group members’ characteristics. Cases with missing data for any of the predictors were excluded from the analyses, which were all repeated using multiple imputation, eliciting no substantial differences in results compared with listwise deletion, as documented in the Supplement (Sensitivity of linear regression results). Data and analysis codes are available online [51].

### Ethical statement

The University of Erfurt’s institutional review board approved this study (Number 20200501), which is in line with the 2013 Declaration of Helsinki. Participants provided informed consent before participating in the study.

## Results

An overview of the study participants across the four rounds of data collection compared to respective distributions of demographic variables in the German population is presented (Table 1).

**Table 1:** Overview of survey participants across four rounds of data collection and respective distributions of demographic variables in the population, Germany, 3–25 March 2020 (n = 3,910)

**Characteristics**	**German population**	**Round 1** **(3–4 Mar)**		**Round 2** **(10–11 Mar)**		**Round 3** **(17–18 Mar)**		**Round 4** **(24–25 Mar)**	
Participants (n)	Census^a^	973		966		1,015		956	
Age in years, mean (SD)	46.1	46.4 (15.6)		46.5 (15.7)		46.3 (15.7)		46.0 (16.0)	
Sex	%	n	%	n	%	n	%	n	%
Men	49.7	491	50.5	461	47.7	506	49.9	495	51.8
Women	50.3	482	49.5	505	52.3	509	50.1	461	48.2
Education^b^									
Low	41.7	106	10.9	102	10.6	121	11.9	96	10.0
Middle	37.3	350	36.0	336	34.8	375	36.9	325	34.0
High	21.1	517	53.1	528	54.7	519	51.1	535	56.0
Community size^c^									
Small town	NA	365	37.5	366	37.9	408	40.2	370	38.7
Medium town	NA	256	26.3	244	25.3	250	24.6	234	24.5
Large town	NA	352	36.2	356	36.9	357	35.2	352	36.8
Employed in the health sector									
Yes	NA	81	8.3	92	9.5	83	8.2	76	7.9
No	NA	892	91.7	874	90.5	932	91.8	880	92.1
Data completeness^d^									
Dropouts	NA	52	5.1	65	6.3	71	6.5	291	23.3

COVID-19: coronavirus disease; COSMO: COVID-19 Snapshot Monitoring; NA: not available or applicable for census data; SD: standard deviation.

^a^ Figures for the German population are based on the 2011 German census.

^b^ Education was assessed as low (COSMO: up to 9 years of schooling; census: no school or no graduation, elementary or a secondary school diploma); middle (COSMO: at least 10 years, without university entrance qualification; census: intermediate graduation and upper secondary school, advanced technical college entrance qualification); or high (COSMO: at least 10 years, with university entrance qualification; census: general or subject-related university entrance qualification).

^c^ Community size: small: < 20,000 inhabitants; medium: 20,000–100,000 inhabitants; large: > 100,000 inhabitants.

^d^ Dropouts comprise the number of participants who withdrew from the survey after they started.

### Descriptive data

**Figure 1:**
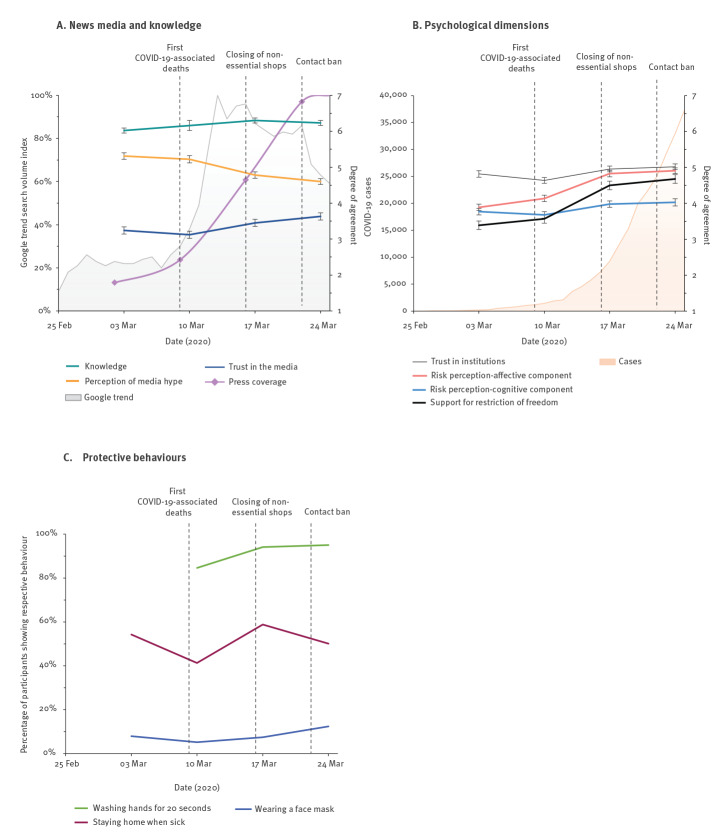
Epidemiological, informational and psychological aspects at the onset of the COVID-19 pandemic in Germany, 25 February–24 March 2020 (n = 3,910)

COSMO: COVID-19 Snapshot Monitoring; COVID-19: coronavirus disease.

Note: Dashed lines indicate major events and policies (first COVID-19–associated death, school and non-essential shop closures, contact bans/lockdowns). Data sources: Behavioural and attitudinal data collected from the COSMO survey, case numbers from Robert Koch Institute, Google Trends data and newspaper volume data from the Wiso press database. Error bars are at 95% confidence intervals.

We found an increasingly active search for information based on Google Trends data and an increasing availability of information in the news media (Figure 1A). Despite the steep increase in news media reporting, the situation was viewed as less of a news media-hyped story as time passed, and trust in media increased correspondingly. SARS-CoV-2- and COVID-19-related knowledge was high during these 4 weeks, and increased between the first and last round of our survey [60]. Knowledge about the lack of medical treatments and vaccines, in particular, increased between the second and third round (Supplementary Table S1: Participants’ characteristics across the time points), the period during which the largest increase in information searches and availability occurred. Interestingly, Google searches peaked relatively soon after the first COVID-19-associated death, reaching further local maxima when new regulations took effect. The cognitive risk perceptions, e.g. regarding susceptibility, increased very slowly (Figure 1B), but affective risk perceptions increased sharply between the first COVID-19-associated death and the closing of non-essential shops and schools. As affective risk perceptions increased, acceptance of restrictive measures increased to a similar extent. Trust in institutions remained high, even after strict measures were implemented, potentially because of the high acceptance of strict measures at that time. Suggested protective behaviours increased over time (Figure 1C).

### Acceptance of restrictive policies

**Figure 2:**
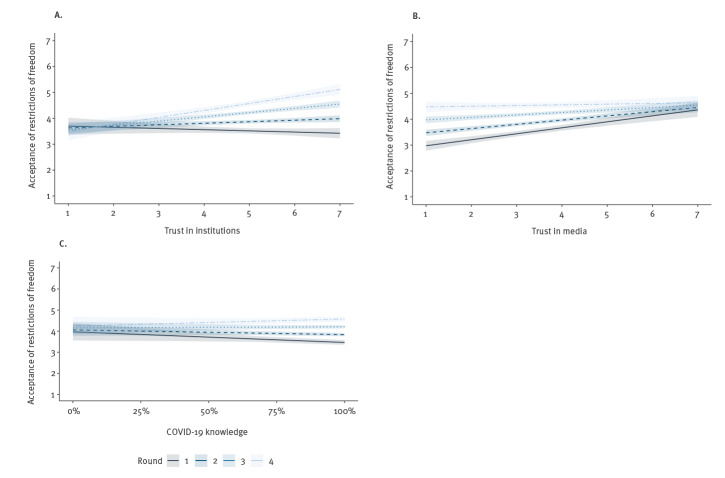
Result of regressing the acceptance of restrictions on trust and knowledge over time, Germany, 3–25 March 2020 (n = 3,910)

When predicting the acceptance of restrictive policies, the addition of psychological variables to the demographics increased the amount of explained variance from 9% to 19% (Table 2). Acceptance of restrictive policies increased with age and employment in the healthcare sector. Both age and employment in the health sector are risk factors, as age increases the likelihood for severe disease and being employed in the health sector increases the risk of being exposed to the virus. Regarding psychological dimensions, both cognitive and affective risk perceptions were significant predictors of acceptance. Generally, acceptance of restrictive measures increased over time and was higher among those with greater trust in institutions and media. However, these effects changed over time, as indicated by several interaction effects. Trust in institutions became more relevant over time (Figure 2A), whereas trust in media became somewhat less relevant over time (Figure 2B). Greater knowledge of COVID-19 was associated with lower acceptance of restrictions early in the outbreak, but this association diminished over time (Figure 2C).

**Table 2:** Results of regressing acceptance of restrictive policies on demographics, time and psychological variables, Germany, 3–25 March 2020 (n = 3,910)

Predictors	Model 1 (demographics and time)			Model 2 (psychological variables)			Model 3 (interactions between psychological variables and time)		
	Beta	CI	p value	Beta	CI	p value	Beta	CI	p value
Age (years)	0.13	0.10 to 0.16	< 0.001	0.07	0.04 to 0.10	< 0.001	0.07	0.04 to 0.10	< 0.001
Occupation in the health sector	0.03	−0.00 to 0.06	0.099	0.04	0.01 to 0.07	0.010	0.04	0.01 to 0.07	0.011
Time (round)	0.28	0.25 to 0.31	< 0.001	0.19	0.16 to 0.22	< 0.001	−0.05	−0.22 to 0.13	0.594
Cognitive component of risk	NA			0.07	0.04 to 0.10	< 0.001	0.12	0.05 to 0.19	0.001
Trust in institutions	NA			0.07	0.03 to 0.10	< 0.001	−0.10	−0.18 to −0.02	0.012
Trust in media	NA			0.11	0.07 to 0.14	< 0.001	0.24	0.16 to 0.32	< 0.001
Affective component of risk	NA			0.23	0.20 to 0.27	< 0.001	0.22	0.19 to 0.26	< 0.001
Self-efficacy	NA			0.03	−0.01 to 0.06	0.120	0.02	−0.01 to 0.05	0.146
COVID-19 knowledge	NA						−0.08	−0.1 to −0.01	0.021
Trust in institutions * time	NA						0.36	0.21 to 0.51	< 0.001
Trust in media * time	NA						−0.21	−0.32 to −0.09	< 0.001
COVID-19 knowledge * time	NA						0.16	0.02 to 0.30	0.026
Cognitive component of risk * time	NA						−0.08	−0.20 to 0.03	0.136
Observations	3,731			3,731			3,731		
R^2^/adjusted R^2^	0.093/0.092			0.187/0.186			0.195/0.192		

CI: confidence interval; COVID-19: coronavirus disease; NA: variable not included in the respective model; R^2^: coefficient of determination.

Variables in the model were age, sex, education, occupation in the health sector, community size, trust in institutions, trust in media, COVID-19 knowledge, knowledge regarding protective behaviour, cognitive component of risk, affective component of risk, self-efficacy, time (round) and the interactions of the psychological variables and time.

The results indicate that trust in institutions (government, health authorities) became more important over time for the acceptance of restrictive measures (panel A), while the importance of trust in media declined over time (panel B). Though greater knowledge decreased compliance at the beginning of the German epidemic, knowledge was rather unrelated with compliance a month later (panel C). Round denotes the round of data collection; ratings were collected on Likert-type scales ranging from 1 to 7.

### Population groups and acceptance

**Figure 3:**
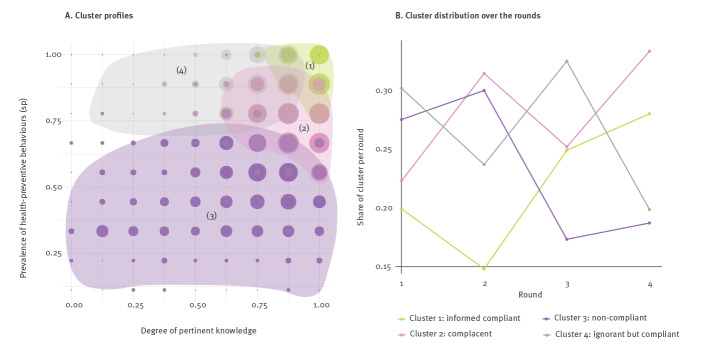
Clusters resulting from differences between knowledge about protective measures and actual behaviours, and variations in size over time, Germany, 3–25 March 2020 (n = 3,910)

Knowledge about effective preventive behaviour is not a guarantee of compliance [47]; therefore, we extracted profiles of homogenous groups of people to examine knowledge and compliance with requested behaviour. On the basis of eight items on pandemic-related knowledge, e.g. knowing that handwashing is an effective preventive measure, and eight items on various protective and crisis-related behaviours, e.g. cancelling planned travels (see the Supplement), four distinct clusters were identified based on results from all four data collection rounds (see Figure 3).

Four clusters resulted from the analysis (left) across all four data collection rounds: Cluster 1: informed compliant, (light green) scored high on both knowledge and behaviour, Cluster 2: complacent (pink) was well-informed, but fell short in acting on their knowledge, Cluster 3: non-compliant (purple) included those practising little or no protective behaviour and contains the largest share of participants with very low knowledge levels and Cluster 4: ignorant but compliant (grey) scored low in knowledge, but nevertheless took appropriate actions to prevent the spread of the virus. Compared with all other clusters, the informed compliant cluster remains relatively small over time. While clusters with high knowledge levels comprise 60% of the sample in round 4, half of the samples are still either not compliant at all or comply less than their knowledge would suggest. A detailed list of the items used and rationale for the number of clusters extracted can be found in the Supplement.

Participants in Cluster 1 are considered ‘informed compliant’ because they score high on both knowledge and behaviour (Figure 3A). Those grouped in Cluster 2, defined as ‘complacent’, can be characterised as well-informed, but falling short in acting on their knowledge about COVID-19. Participants in Cluster 3 are regarded as ‘non-compliant’, which features those practising little or no protective behaviours. This cluster also contains the largest share of participants with very low knowledge levels. Participants in Cluster 4, classed as ‘ignorant but compliant’, scored low in knowledge, but nevertheless took appropriate actions to prevent the spread of the virus. At the beginning of the pandemic, more than half the participants belonged to the two clusters scoring low in knowledge (Clusters 3: non-compliant and 4: ignorant but compliant) (Figure 3B). The share of people in these two clusters decreased over time. The share of the ‘informed compliant’ cluster increased significantly over time, while the share of the ‘non-compliant’ cluster decreased sharply. At the end of data collection, around 60% of the participants were in the two clusters scoring high on knowledge. Even so, the clusters that were not fully compliant with appropriate measures still comprised around half of all participants. It is noteworthy that among those who did not comply, the share of those who also lacked knowledge was relatively small.

In the next step, we examined which variables explained cluster membership using a multinomial logistic regression approach. We used time (round), sociodemographic and psychological factors as sets of predictors in a full model (entering all sets simultaneously) and in single models (entering only one set) (Table 3). The analysis revealed that being older increased preventive behaviours and high knowledge levels, i.e. the likelihood of being in the ‘informed compliant’ cluster. Notably, having a higher education level increased the gap between knowledge and behaviour, in that higher education was related positively to being in the ‘non-compliant’ or ‘ignorant but compliant’ cluster. Cognitive and affective risk perceptions were related to showing more protective behaviour, which is indicated by the negative associations with belonging to the ‘non-compliant’ cluster or the ‘complacent’ cluster. Trust in institutions was related to turning knowledge into action, as trust was related negatively with belonging to the ‘non-compliant’ cluster.

**Table 3:** Multinomial logistic regression predicting cluster membership, displaying log odds for the full model and single sets of variables, Germany, 3–25 March 2020 (n = 3,910)

**Category^a^**	**Full model**			**Single models**		
	**Cluster 2:** **complacent**	**Cluster 3:** **non-compliant**	**Cluster 4:** **ignorant but compliant**	**Cluster 2:** **complacent**	**Cluster 3:** **non-compliant**	**Cluster 4:** **ignorant but compliant**
Intercept	1.938*** (0.339)	3.854*** (0.375)	1.214*** (0.345)	0.417*** (0.119)	0.982*** (0.121)	0.812*** (0.117)
Time	0.034 (0.044)	−0.234*** (0.049)	−0.195*** (0.045)	−0.043 (0.041)	−0.409*** (0.045)	−0.234*** (0.042)
**Sociodemographic variables**						
Intercept	NA			0.480*** (0.186)	0.314 (0.199)	0.289 (0.190)
Age	−0.003 (0.003)	−0.004 (0.003)	−0.006** (0.003)	−0.006** (0.003)	−0.008** (0.003)	−0.007** (0.003)
Higher education: Yes	0.071 (0.096)	0.154 (0.107)	0.197** (0.099)	0.110 (0.095)	0.176* (0.102)	0.215** (0.097)
Sex: Female	0.049 (0.093)	−0.065 (0.104)	0.245** (0.095)	0.001 (0.091)	−0.186* (0.098)	0.188** (0.093)
Occupation health sector: Yes	0.025 (0.160)	−0.687*** (0.203)	−0.161 (0.168)	0.116 (0.158)	−0.515*** (0.195)	−0.094 (0.165)
Community size^b^: Medium town	0.092 (0.117)	0.051 (0.131)	−0.037 (0.120)	0.102 (0.115)	0.105 (0.125)	−0.018 (0.119)
Community size^b^: Large town	0.106 (0.108)	0.163 (0.119)	0.116 (0.109)	0.087 (0.106)	0.140 (0.114)	0.103 (0.107)
**Psychological variables**						
Intercept	NA			2.027*** (0.304)	3.560*** (0.333)	1.096*** (0.307)
Trust in institutions	0.054 (0.036)	−0.138*** (0.039)	0.055 (0.037)	0.050 (0.036)	−0.143*** (0.038)	0.050 (0.036)
Cognitive risk	−0.191*** (0.035)	−0.169*** (0.040)	−0.017 (0.036)	−0.196*** (0.035)	−0.174*** (0.039)	−0.029 (0.035)
Affective risk	−0.130*** (0.039)	−0.378*** (0.043)	−0.137*** (0.040)	−0.125*** (0.037)	−0.434*** (0.041)	−0.184*** (0.038)
Self-efficacy	−0.135*** (0.037)	−0.054 (0.042)	−0.002 (0.038)	−0.141*** (0.036)	−0.083** (0.041)	−0.035 (0.037)
**Goodness of fit**						
AIC	10,209.020			Time: 10,664.130 Sociodemographic variables: 10,755.330 Psychological factors: 10,268.810		
Nagelkerke’s pseudo R^2^	0.104			Time: 0.031 Sociodemographic variables: 0.011 Psychological factors: 0.078		

AIC: Akaike information criterion. NA: not applicable in the full model.

^a^ Reference is Cluster 1: informed compliant.

^b^ Community size: small: < 20,000 inhabitants; medium: 20,000–100,000 inhabitants; large: > 100,000 inhabitants.

Four clusters resulted from the analysis across all four data collection rounds (Figure 3): Cluster 1: informed compliant scored high on both knowledge and behaviour, Cluster 2: complacent was well-informed, but fell short in acting on their knowledge, Cluster 3: non-compliant included those practising little or no protective behaviour and contains the largest share of participants with very low knowledge levels and Cluster 4: ignorant but compliant scored low in knowledge, but nevertheless took appropriate actions to prevent the spread of the virus. Table displays the estimated multinomial logistic regression coefficients and standard errors in brackets. Significance values are as follows: *p < 0.1; **p < 0.05; ***p < 0.01.

## Discussion

Published studies on protective behaviour during the COVID-19 pandemic have demonstrated that motivation and knowledge are relevant drivers [61]. However, most of these studies took place at a later stage in the pandemic and collected data only once, sometimes twice, which does not allow understanding of change over time [3-27]. As early action is critical, given uncertain and potentially drastic consequences [48], it is of utmost importance to learn how people adapt to such a situation so that policymakers are more informed regarding how to guide people’s behaviour effectively.

The data reported here are unique, as they were collected weekly during the 4-week period from the report of the first COVID-19 cases in Germany to the first national lockdown. Our data indicated that public perceptions and opinions adapted rapidly to the new threat and to the need for radical behavioural changes, which were partially regulated by strict policies mandated by the German government. The dynamic spread of COVID-19 was mirrored by affective risk data; feelings of fear and being at risk increased sharply at the outset and were more closely related to acceptance of strict measures rather than perceived susceptibility to contracting the disease. Other published studies from the same time period also indicate that perceiving governmental actions as non-efficacious was related to increased worry and fear [26]. Thus, while facing drastic measures may be a cue that elicits fear, the lockdown also may have led to a containment of fear [31] and an increase in trust. As the pandemic progresses further, it will be relevant to assess how adaptation to the threat and decreasing risk perceptions affect the acceptance of strict measures and trust in government authorities.

Our data reveal that trust in institutions played an increasing role in the acceptance of measures as cases increased and more restrictive measures were implemented. Thus, at these critical points in time, German health authorities and the government seem to have acted in ways that strengthened trust. Not only did the relationship between trust and acceptance of measures increase, but so did average trust itself. This suggests that trust will remain a crucial factor as the pandemic continues. Trust can be achieved through transparency and the use of available evidence [62]. However, evidence changes quickly during such a dynamic situation [63]. Meta-communication about how knowledge changes and how this knowledge is still used to update policies may increase trust in acting authorities.

News media have been an important source of information, especially at the beginning of the pandemic and when new restrictions first came into effect. As data from Italy indicate, consumption of more media reports was also related to higher risk perceptions [30]. In addition, although news media are an important knowledge source, nearly half the study’s participants were not fully compliant when the lockdown was implemented, despite high knowledge levels. Interestingly, participants with higher education levels fell into the two clusters in which knowledge and behaviour were not related, e.g. ‘non-compliant’ and ‘ignorant but compliant’. More highly educated individuals may have more freedom to choose whether they comply with rules, possibly because they were less likely to work in frontline jobs involving situations with increased risk of SARS-CoV-2 infection [64,65]. Furthermore, we found that women’s display of protective behaviours was unrelated to knowledge, and those who felt they were at lower risk practised less protective behaviours, regardless of knowledge. Men tended to be non-compliant, regardless of knowledge. Findings from other countries also suggest that men were a main target group for interventions as they showed less preventive behaviours [31,66]. Younger people were also an important group to assess because they were less likely to be in the ‘informed compliant’ Cluster 1, possibly because of high knowledge but lower risk perceptions and thus lower motivation to protect themselves.

The four clusters described can be used to identify target groups for further interventions. Inspecting potential levers suggests that strategies addressing affect may be useful because feeling at risk was a relevant difference between those categorised as ‘informed compliant’ and the other clusters. However, it is important to note that these findings do not mean that appeals to fear are the solution to promoting compliance [67]. Eliciting strong fear may erode trust and create reactance, i.e. a state of anger and counter-reaction [68]. Strategies that increase self-efficacy in complacent people could increase protective behaviour and help turn knowledge into action. Recommendations from the analyses are summarised in the Box.

Box **Recommendations for healthcare authorities**

**Data collection**

- Install a representative population panel that enables rapid surveillance of psychological factors that can affect acceptance of measures at the early stages of a pandemic (or other major health crisis) to identify target groups for health communication and other interventions.

**Health communication**

- Install a mechanism through which to communicate results to different audiences in a timely and understandable fashion, e.g. to national and local crisis teams, politicians, news media and the general public.
- Provide information, e.g. about how a pathogen is transmitted, to allow for informed compliance, e.g. protective behaviour. Delivering relevant knowledge is necessary, but insufficient to elicit desired behaviour.
- Increase awareness of the social impact of non-compliance in order to increase the motivation of well-informed but non-compliant individuals. Although feeling at least somewhat at risk is necessary, communicating daily case numbers may not be sufficient, as perceived susceptibility was less important. Be wary of using strong fear appeals, as they can backfire and increase opposition to measures. If risks are pointed out, provide information to increase self-efficacy, i.e. empower people to protect themselves and others.
- Knowledge–behaviour gaps may be particularly high among the well-educated. Identify target groups and whether these groups lack knowledge or motivation to comply.
- High trust can turn knowledge into action, but trust may change over time, particularly during crises with long durations. Thus, maintaining trust is vital. Changing policies can increase trust if risk perceptions are high, and the measures are viewed as appropriate. However, changing policies can also create challenges in preserving trust. Highlighting that decisions are evidence-based, and that evidence changes often, can be helpful.

**Outreach**

- Find ways to boost compliance. For example, if women comply regardless of knowledge, encourage women in campaigns to help their family and friends. If elderly people comply based on high knowledge, motivate them to explain their reasons for compliance to their families.
- Cluster analyses can be used to identify important sources of information to ensure optimal outreach from communication campaigns.

This study has some limitations. Firstly, our estimates of article output containing ‘corona’ did not exclude double mentions, which may be due to different editions of local newspapers. However, the data provide an estimate of the topic’s presence in news media. Secondly, the survey data were collected online. We refrained from including people over 75 years of age because coverage bias for online surveys in this age group may be particularly prominent [69], and confounders that might affect health could come into play, e.g. more active lifestyles in older adults who use the internet vs non-users [70]. We also refrained from including people under 18 years of age, as parental consent would have been necessary. Thirdly, other work has documented psychological strain and the consequences of the pandemic for children and adolescents [71,72], but this was beyond the scope of this study. Fourthly, our study also relied on self-reporting, which may be subject to social desirability bias [73], considering that individuals with high social desirability may not only report more compliant behaviour, but may also actually be more compliant when it is currently socially desirable to do so. Finally, the statistical analyses are unweighted [58]. As the sample’s characteristics deviated from the general population in terms of education and age, this potentially inflates the sizes of the ‘non-compliant’ and ‘ignorant but compliant’ clusters. 

In general, the repeated cross-sectional data do not permit causal inferences, but they still permit important insights into changes in average perceptions and behaviour over time, given that participants in each group were sampled randomly from the same population. Moreover, non-probability online surveys were the only viable method available during the lockdown period for gathering data in a timely manner; personal interviews were precluded by the risk of transmission, phone studios for computer-assisted telephone interviewing surveys were closed and available probability-based survey infrastructures either were restricted to certain research groups or did not provide sufficient capacities to accommodate this study. Multiple reasons may explain the response rates of 15–20% for contacted individuals. A large number of participants matching the quotas had to be recruited during a very short period of time. This required inviting larger groups, as people may not have seen the invitation in their email inbox or could not find time to answer within the very short time frame. Moreover, response rates decreased with age and were lower in small federal states [74], which may have lowered the overall response rate. It remains possible that individuals with particular interest in the topic are overrepresented, but this limitation pertains to all survey methods.

## Conclusion

As the COVID-19 pandemic continues, knowledge about acceptance factors that drive individual protective behaviour, such as physical distancing, is crucial to further support strategies that help adjust behaviour to control the pandemic. It is important to note that such data always will be a snapshot with many confounding factors, such as news media reports, political discussions and decisions, case numbers and deaths. This naturally impedes causal interpretations of changes over time. If resources allow, a complementary longitudinal study design could add important insights. Nevertheless, the current study provides colleagues in other countries with a blueprint for analysing, interpreting and using the data collected in other countries (Box). This may help identify cross-national commonalities and differences relevant to public health measures aimed at controlling this pandemic, as well as future outbreaks of new pathogens.

## Supplementary Data

4650000 Supplement

## References

[1] Haug N Geyrhofer L Londei A Dervic E Desvars-Larrive A Loreto V Ranking the effectiveness of worldwide COVID-19 government interventions. Nat Hum Behav. 2020;4(12):1303-12. 10.1038/s41562-020-01009-0 33199859

[2] Tian H Liu Y Li Y Wu C-H Chen B Kraemer MUG An investigation of transmission control measures during the first 50 days of the COVID-19 epidemic in China. Science. 2020;368(6491):638-42. 10.1126/science.abb6105 32234804 PMC7164389

[3] Bruine de Bruin W. Age differences in COVID-19 risk perceptions and mental health: evidence from a national U.S. survey conducted in March 2020. J Gerontol B Psychol Sci Soc Sci. 2021;76(2):e24-9. 10.1093/geronb/gbaa074 32470120 PMC7542924

[4] Bruine de Bruin W Bennett D. Relationships between initial Covid-19 risk perceptions and protective health behaviors: a national survey. Am J Prev Med. 2020;59(2):157-67. 10.1016/j.amepre.2020.05.001 32576418 PMC7242956

[5] Masters NB Shih S-F Bukoff A Akel KB Kobayashi LC Miller AL Social distancing in response to the novel coronavirus (COVID-19) in the United States. PLoS One. 2020;15(9):e0239025. 10.1371/journal.pone.0239025 32915884 PMC7485770

[6] McFadden SM Malik AA Aguolu OG Willebrand KS Omer SB. Perceptions of the adult US population regarding the novel coronavirus outbreak. PLoS One. 2020;15(4):e0231808. 10.1371/journal.pone.0231808 32302370 PMC7164638

[7] Lennon RP Sakya SM Miller EL Snyder B Yaman T Zgierska AE Public intent to comply with covid-19 public health recommendations. Health Lit Res Pract. 2020;4(3):e161-5. 10.3928/24748307-20200708-01 32926171 PMC7410495

[8] Ciancio A Kämpfen F Kohler IV Bennett D Bruine de Bruin W Darling J Know your epidemic, know your response: Early perceptions of COVID-19 and self-reported social distancing in the United States. PLoS One. 2020;15(9):e0238341. 10.1371/journal.pone.0238341 32886671 PMC7473541

[9] Duan T Jiang H Deng X Zhang Q Wang F. Government intervention, risk perception, and the adoption of protective action recommendations: evidence from the covid-19 prevention and control experience of China. Int J Environ Res Public Health. 2020;17(10):3387. 10.3390/ijerph17103387 32414013 PMC7277925

[10] Ding Y Xu J Huang S Li P Lu C Xie S. Risk perception and depression in public health crises: evidence from the covid-19 crisis in China. Int J Environ Res Public Health. 2020;17(16):5728. 10.3390/ijerph17165728 32784792 PMC7460398

[11] He S Chen S Kong L Liu W. Analysis of risk perceptions and related factors concerning covid-19 epidemic in Chongqing, China. J Community Health. 2021;46(2):278-85. 32592160 10.1007/s10900-020-00870-4 PMC7318903

[12] Huynh TLD. Data for understanding the risk perception of COVID-19 from Vietnamese sample. Data Brief. 2020;30:105530. 10.1016/j.dib.2020.105530 32322641 PMC7171448

[13] Bodrud-Doza M Shammi M Bahlman L Islam ARMT Rahman MM. Psychosocial and socio-economic crisis in Bangladesh due to covid-19 pandemic: a perception-based assessment. Front Public Health. 2020;8:341. 10.3389/fpubh.2020.00341 32676492 PMC7333562

[14] Harapan H Anwar S Nainu F Setiawan AM Yufika A Winardi W Perceived risk of being infected with SARS-CoV-2: a perspective from Indonesia. Disaster Med Public Health Prep. 2020;1-5. 10.1017/dmp.2020.351 32907679 PMC7674822

[15] Reddy SP Sewpaul R Mabaso M Parker S Naidoo I Jooste S South Africans’ understanding of and response to the COVID-19 outbreak: an online survey. S Afr Med J. 2020;110(9):894-902. 10.7196/SAMJ.2020.v110i9.14838 32880275

[16] Olapegba PO Iorfa SK Kolawole SO Oguntayo R Gandi JC Ottu IFA Survey data of COVID-19-related knowledge, risk perceptions and precautionary behavior among Nigerians. Data Brief. 2020;30:105685. 10.1016/j.dib.2020.105685 32391411 PMC7206440

[17] Sengeh P Jalloh MB Webber N Ngobeh I Samba T Thomas H Community knowledge, perceptions and practices around COVID-19 in Sierra Leone: a nationwide, cross-sectional survey. BMJ Open. 2020;10(9):e040328. 10.1136/bmjopen-2020-040328 32948576 PMC7500298

[18] Seale H Heywood AE Leask J Sheel M Thomas S Durrheim DN COVID-19 is rapidly changing: Examining public perceptions and behaviors in response to this evolving pandemic. PLoS One. 2020;15(6):e0235112. 10.1371/journal.pone.0235112 32574184 PMC7310732

[19] Germani A Buratta L Delvecchio E Mazzeschi C. Emerging adults and covid-19: the role of individualism-collectivism on perceived risks and psychological maladjustment. Int J Environ Res Public Health. 2020;17(10):3497. 10.3390/ijerph17103497 32429536 PMC7277425

[20] Motta Zanin G Gentile E Parisi A Spasiano D. A preliminary evaluation of the public risk perception related to the covid-19 health emergency in Italy. Int J Environ Res Public Health. 2020;17(9):3024. 10.3390/ijerph17093024 32349253 PMC7246845

[21] Gesser-Edelsburg A Cohen R Hijazi R Abed Elhadi Shahbari N. Analysis of public perception of the Israeli government’s early emergency instructions regarding covid-19: online survey study. J Med Internet Res. 2020;22(5):e19370. 10.2196/19370 32392172 PMC7236609

[22] Losada-Baltar A Jiménez-Gonzalo L Gallego-Alberto L Pedroso-Chaparro MDS Fernandes-Pires J Márquez-González M. We are staying at home: association of self-perceptions of aging, personal and family resources, and loneliness with psychological distress during the lock-down period of covid-19. J Gerontol B Psychol Sci Soc Sci. 2021;76(2):e10-16. 10.1093/geronb/gbaa048 32282920 PMC7184373

[23] Abdelhafiz AS Mohammed Z Ibrahim ME Ziady HH Alorabi M Ayyad M Knowledge, perceptions, and attitude of Egyptians towards the novel coronavirus disease (COVID-19). J Community Health. 2020;45(5):881-90. 10.1007/s10900-020-00827-7 32318986 PMC7173684

[24] Shiina A Niitsu T Kobori O Idemoto K Hashimoto T Sasaki T Relationship between perception and anxiety about COVID-19 infection and risk behaviors for spreading infection: A national survey in Japan. Brain Behav Immun Health. 2020;6:100101. 10.1016/j.bbih.2020.100101 32835297 PMC7331545

[25] Geldsetzer P. Use of rapid online surveys to assess people's perceptions during infectious disease outbreaks: a cross-sectional survey on COVID-19. J Med Internet Res. 2020;22(4):e18790. 32240094 10.2196/18790 PMC7124956

[26] Mækelæ MJ Reggev N Dutra N Tamayo RM Silva-Sobrinho RA Klevjer K Perceived efficacy of COVID-19 restrictions, reactions and their impact on mental health during the early phase of the outbreak in six countries. R Soc Open Sci. 2020;7(8):200644. 10.1098/rsos.200644 32968525 PMC7481706

[27] Vally Z. Public perceptions, anxiety and the perceived efficacy of health-protective behaviours to mitigate the spread of the SARS-Cov-2/ COVID-19 pandemic. Public Health. 2020;187:67-73. 10.1016/j.puhe.2020.08.002 32927291 PMC7414382

[28] Reynolds B W Seeger M. Crisis and emergency risk communication as an integrative model. J Health Commun. 2005;10(1):43-55. 10.1080/10810730590904571 15764443

[29] Rasmussen SA, Goodman RA. The CDC field epidemiology manual. Oxford: Oxford University Press; 2018.

[30] Rubaltelli E Tedaldi E Orabona N Scrimin S. Environmental and psychological variables influencing reactions to the COVID-19 outbreak. Br J Health Psychol. 2020;25(4):1020-38. 10.1111/bjhp.12473 32951244 PMC7537169

[31] Abir T Kalimullah NA Osuagwu UL Yazdani DMN Mamun AA Husain T Factors associated with the perception of risk and knowledge of contracting the SARS-Cov-2 among adults in Bangladesh: analysis of online surveys. Int J Environ Res Public Health. 2020;17(14):5252. 10.3390/ijerph17145252 32708161 PMC7400220

[32] Marinthe G Brown G Delouvée S Jolley D. Looking out for myself: Exploring the relationship between conspiracy mentality, perceived personal risk, and COVID-19 prevention measures. Br J Health Psychol. 2020;25(4):957-80. 10.1111/bjhp.12449 32583540 PMC7361332

[33] Reintjes R Das E Klemm C Richardus JH Keßler V Ahmad A. ”Pandemic public health paradox”: time series analysis of the 2009/10 influenza A/H1N1 epidemiology, media attention, risk perception and public reactions in 5 European countries. PLoS One. 2016;11(3):e0151258. 10.1371/journal.pone.0151258 26982071 PMC4794201

[34] Betsch C Wieler LH Habersaat K COSMO group. Monitoring behavioural insights related to COVID-19. Lancet. 2020;395(10232):1255-6. 10.1016/S0140-6736(20)30729-7 32247323 PMC7163179

[35] COSMO Group. Covid-19 snapshot monitoring. Trier: PsychArchives. [Accessed: 29 Sep 2021]. 10.23668/psycharchives.2776

[36] World Health Organization Regional Office for Europe (WHO/Euro). COVID-19 Snapshot Monitoring (COSMO Standard): Monitoring knowledge, risk perceptions, preventive behaviours, and public trust in the current coronavirus outbreak - WHO standard protocol. Copenhagen: WHO/Euro. [Accessed: 29 Sep 2021]. Available from: https://www.psycharchives.org/handle/20.500.12034/2392

[37] World Health Organization (WHO). Survey tool and guidance: rapid, simple, flexible behavioural insights on COVID-19: 29 July 2020. Geneva: WHO; 2020. Available from: https://apps.who.int/iris/handle/10665/333549

[38] Gupta N Fischer ARH Frewer LJ. Socio-psychological determinants of public acceptance of technologies: A review. Public Underst Sci. 2012;21(7):782-95. 10.1177/0963662510392485 23832558 PMC3546631

[39] Rubin GJ Amlôt R Page L Wessely S. Public perceptions, anxiety, and behaviour change in relation to the swine flu outbreak: cross sectional telephone survey. BMJ. 2009;339:b2651. 10.1136/bmj.b2651 19574308 PMC2714687

[40] May PJ. Regulation and compliance motivations: examining different approaches. Public Adm Rev. 2005;65(1):31-44. 10.1111/j.1540-6210.2005.00428.x

[41] Feufel MA Antes G Gigerenzer G. Vom sicheren Umgang mit Unsicherheit: Was wir von der pandemischen Influenza (H1N1) 2009 lernen können. [Dealing safely with uncertainty: what we can learn from pandemic influenza (H1N1) 2009]. Bundesgesundheitsblatt. 2010;53(12):1283-9. German. 10.1007/s00103-010-1165-1 21161479

[42] van der Pligt J. Risk perception and self-protective behavior. Eur Psychol. 1996;1(1):34-43. 10.1027/1016-9040.1.1.34

[43] Earle TC, Siegrist M, Gutscher H. Trust, Risk Perception and the TGC Model of Cooperation. In: Trust in Risk Management. London: Routledge, 2010. S. 18-66.

[44] Brugger J Hawkes KL Bowen AM McClaran MP. Framework for a collaborative process to increase preparation for drought on U.S. public rangelands. Ecol Soc. 2018;23(4):18. 10.5751/ES-10503-230418

[45] Rogers RW. A protection motivation theory of fear appeals and attitude change1. J Psychol. 1975;91(1):93-114. 10.1080/00223980.1975.9915803 28136248

[46] Becker MH. The health belief model and sick role behavior. Health Educ Monogr. 1974;2(4):409-19. 10.1177/109019817400200407

[47] Rimal RN. Closing the knowledge-behavior gap in health promotion: the mediating role of self-efficacy. Health Commun. 2000;12(3):219-37. 10.1207/S15327027HC1203_01 10938914

[48] Abou Chakra M Bumann S Schenk H Oschlies A Traulsen A. Immediate action is the best strategy when facing uncertain climate change. Nat Commun. 2018;9(1):2566. 10.1038/s41467-018-04968-1 29967461 PMC6028488

[49] Robert Koch-Institut (RKI). Robert Koch-Institut: COVID-19-Dashboard. Berlin: RKI. [Accessed: 21 Apr 2021]. German. Available from: https://experience.arcgis.com/experience/478220a4c454480e823b17327b2bf1d4

[50] International Organization for Standardization (ISO). Access panels in market, opinion and social research — vocabulary and service requirements. ISO/IEC 26362:2009. Geneva: ISO; 2009. Available from: https://www.iso.org/standard/43521.html

[51] Betsch C, Bosnjak M, Korn L, Burgard T, Gaissmaier W. Supplementary materials to: The four weeks before lockdown during the COVID-19 pandemic in Germany. PsychArchives. 2021. 10.23668/PSYCHARCHIVES.4786 PMC8532505 34676821

[52] Brewer NT Chapman GB Gibbons FX Gerrard M McCaul KD Weinstein ND. Meta-analysis of the relationship between risk perception and health behavior: the example of vaccination. Health Psychol. 2007;26(2):136-45. 10.1037/0278-6133.26.2.136 17385964

[53] Bradley MM Lang PJ. Measuring emotion: The self-assessment manikin and the semantic differential. J Behav Ther Exp Psychiatry. 1994;25(1):49-59. 10.1016/0005-7916(94)90063-9 7962581

[54] Google. Google Trends. Mountain View: Google. [Accessed: 19 Oct 2021]. Available from: https://trends.google.com

[55] Zarocostas J. How to fight an infodemic. Lancet. 2020;395(10225):676. 10.1016/S0140-6736(20)30461-X 32113495 PMC7133615

[56] Mheidly N Fares J. Leveraging media and health communication strategies to overcome the COVID-19 infodemic. J Public Health Policy. 2020;41(4):410-20. 10.1057/s41271-020-00247-w 32826935 PMC7441141

[57] Taherdoost H. Determining sample size: how to calculate survey sample size. International Journal of Economics and Management Systems. 2017;2. Available at SSRN: https://ssrn.com/abstract=3224205

[58] Gelman A. Struggles with survey weighting and regression modeling. Stat Sci. 2007;22(2):153-164. 10.1214/088342306000000691

[59] R Core Team. R: a language and environment for statistical computing. R Foundation for Statistical Computing. Vienna: R Core Team; 2016. Available from: https://www.R-project.org

[60] Cumming G. Inference by eye: reading the overlap of independent confidence intervals. Stat Med. 2009;28(2):205-20. 10.1002/sim.3471 18991332

[61] Gibson Miller J Hartman TK Levita L Martinez AP Mason L McBride O Capability, opportunity, and motivation to enact hygienic practices in the early stages of the COVID-19 outbreak in the United Kingdom. Br J Health Psychol. 2020;25(4):856-64. 10.1111/bjhp.12426 32415918 PMC7276910

[62] Aven T Renn O. Improving government policy on risk: eight key principles. Reliab Eng Syst Saf. 2018;176:230-41. 10.1016/j.ress.2018.04.018

[63] Tannert C Elvers HD Jandrig B. The ethics of uncertainty. In the light of possible dangers, research becomes a moral duty. EMBO Rep. 2007;8(10):892-6. 10.1038/sj.embor.7401072 17906667 PMC2002561

[64] Nivette A Ribeaud D Murray A Steinhoff A Bechtiger L Hepp U Non-compliance with COVID-19-related public health measures among young adults in Switzerland: Insights from a longitudinal cohort study. Soc Sci Med. 2021;268:113370. 10.1016/j.socscimed.2020.113370 32980677 PMC7493799

[65] Webster RK Brooks SK Smith LE Woodland L Wessely S Rubin GJ. How to improve adherence with quarantine: rapid review of the evidence. Public Health. 2020;182:163-9. 10.1016/j.puhe.2020.03.007 32334182 PMC7194967

[66] Brouard S Vasilopoulos P Becher M. Sociodemographic and psychological correlates of compliance with the covid-19 public health measures in France. Can J Polit Sci. 2020;53(2):253-8. 10.1017/S0008423920000335

[67] Ruiter RAC Kessels LTE Peters G-JY Kok G. Sixty years of fear appeal research: current state of the evidence. Int J Psychol. 2014;49(2):63-70. 10.1002/ijop.12042 24811876

[68] Brehm JW. Control, its loss, and psychological reactance. In: Control motivation and social cognition. New York: Springer; 1993.

[69] Bosnjak M Dannwolf T Enderle T Schaurer I Struminskaya B Tanner A Establishing an open probability-based mixed-mode panel of the general population in Germany: the GESIS panel. Soc Sci Comput Rev. 2018;36(1):103-15. 10.1177/0894439317697949

[70] Weber W Reinhardt A Rossmann C. Lifestyle segmentation to explain the online health information–seeking behavior of older adults: representative telephone survey. J Med Internet Res. 2020;22(6):e15099. 10.2196/15099 32530433 PMC7320311

[71] Gruber J Prinstein MJ Clark LA Rottenberg J Abramowitz JS Albano AM Mental health and clinical psychological science in the time of COVID-19: challenges, opportunities, and a call to action. Am Psychol. 2021;76(3):409-26. 32772538 10.1037/amp0000707 PMC7873160

[72] Francisco R Pedro M Delvecchio E Espada JP Morales A Mazzeschi C Psychological Symptoms and Behavioral Changes in Children and Adolescents During the Early Phase of COVID-19 Quarantine in Three European Countries. Front Psychiatry. 2020;11:570164. 10.3389/fpsyt.2020.570164 33343415 PMC7744455

[73] Paulhus D. Social desirable responding: the evolution of a construct, pp 49-69. In Braun HI, Jackson DN (Eds.). The role of constructs in psychological and education measurement. Mahwah, New Jersey: Earlbaum Publishers; 2002.

[74] Respondi.de. Panel book. Köln: Respondi.de. [Accessed: 29 Sep 2021]. Available from: https://www.respondi.com/EN/access-panel#download-form

