# Supplementary material for: The four weeks before lockdown during the COVID-19 pandemic in Germany: a weekly serial cross-sectional survey on risk perceptions, knowledge, public trust and behaviour, 3 to 25 March 2020
Source: Euro Surveill. 2021 Oct 21;26(42):2001900. doi: 10.2807/1560-7917.ES.2021.26.42.2001900 (PMC8532505; doi:10.2807/1560-7917.ES.2021.26.42.2001900)
Supplement: Supplement [file 20-01900_BETSCH_supplement.pdf]

Supplementary Appendix

Supplement to

“The four weeks before lockdown during the COVID-19 pandemic in Germany: A weekly serial cross-sectional study on risk perceptions, knowledge, public trust and behaviours in March 2020”

Disclaimer: This supplementary material is hosted by Eurosurveillance as supporting information alongside the article “The four weeks until lockdown during the COVID-19 pandemic in Germany: A weekly serial cross-sectional study on risk perceptions, knowledge, public trust, and behaviours”, on behalf of the authors, who remain responsible for the accuracy and appropriateness of the content. The same standards for ethics, copyright, attributions and permissions as for the article apply. Supplements are not edited by Eurosurveillance and the journal is not responsible for the maintenance of any links or email addresses provided therein.

Supplementary methods **4**
Measures *4*
Demographic variables 4
Trust 4
COVID-19 related knowledge 4
Knowledge about protective behaviours 4
Cognitive risk perception 4
Affective risk perception 4
Disease perceptions 4
Self-efficacy 5
Perception of the outbreak as a media-hype 5
Protective and crisis-related behaviours 5
Time 5
Google trends search volume 5
Newspaper volume data 5
Participants *5*
Supplementary results **6**
Predicting acceptance of measures – assumptions of linear regressions *6*
Assumption 1: Specificity - Linearity 6
Assumption 2: No endogeneity 6
Assumption 3: Homoscedasticity 6
Assumption 4: No autocorrelation 6
Assumption 5: Residuals are normally distributed 6
Assumption 6: No perfect multicollinearity 7
Sensitivity of linear regression results 7
Supplementary tables **9**
Supplementary table 1. Participants' characteristics across the time points. *9*
Supplementary table 2. Results from the Rainbow test. *11*
Supplementary table 3. Correlation table of selected predictors of the models with residuals. *12*
Supplementary table 4. Results of the Goldfeld-Quandt-Tests *13*
Supplementary table 5. Results of the Harrison-McCabe-Tests *14*
Supplementary table 6. Results of the Durbin-Watson-Tests *15*
Supplementary table 7. Correlation table between affective component of risk and perceived distance and spreading of the disease. *16*
Supplementary table 8. Correlation table between trust in institutions and media and knowledge regarding the disease and effective preventive behaviours. *17*
Supplementary table 9. Variance inflation Model 3. .... *18*
Supplementary table 10. Model 1 - Ordinal Logistic vs. Linear Model *19*
Supplementary table 11. Model 2 - Ordinal Logistic vs. Linear Model *20*
Supplementary table 12. Model 3 - Ordinal Logistic vs. Linear Model *21*
Supplementary table 13. Variables used in the cluster analysis *22*
Supplementary table 14. Cluster characteristics *23*

Supplementary figures **24**

Supplementary Figure 1. Scatterplot displaying the relationship between residuals and affective (A) and cognitive (B) risks *24*

Supplementary Figure 2. Scatterplot displaying the relationship between residuals and predicted values in Model 1 (A), Model 2 (B), and Model 3 (C) *25*

Supplementary Figure 3. Histograms displaying the distribution of residuals in Model 1 (A), Model 2 (B), and Model 3 (C) *26*

Supplementary Figure 4. Quantile-quantile-plots displaying theoretical and sample quantiles in Model 1 (A), Model 2 (B), and Model 3 (C) *27*

Supplementary Figure 5. Visualising the intersections of missing values in the data set *28*

Supplementary Figure 6. Visualising the pattern of missing values in the data set *29*

Supplementary Figure 7. Visualising the differences in the estimates (A) and standard errors (B) in Model 1 when deleting missing values vs imputing them *30*

Supplementary Figure 8. Visualising the differences in the estimates (A) and standard errors (B) in Model 2 when deleting missing values vs imputing them *31*

Supplementary Figure 9. Visualising the differences in the estimates (A) and standard errors (B) in Model 3 when deleting missing values vs imputing them *32*

Supplementary Figure 10. Visualising the distribution of (A and B) and the relationship between (C) knowledge regrading protective behaviours and showing protection behaviours. *33*

Supplementary Figure 11. Visualising the optimal numbers of clusters k based on the silhouette method. *34*

Supplementary Figure 12. Visualising the optimal numbers of clusters k based on the total within sum of squares. *35*

Supplementary Figure 12. Clusters resulting from differences in knowledge about protective measures and actual behaviour and their variation in size over time. *36*

Supplementary references **37**

Supplementary methods

Data were collected on March 03/04 (wave 1), 10/11 (wave 2), 17/18 (wave 3), and 24/25 (wave 4), 2020. At the onset of each data collection wave, participants received a link to the online questionnaire and completed it at their own pace; participating took between 15 and 25 minutes (Table 1). At each wave, the same core questions assessing demographics and psychological variables were included in the survey. Original questionnaires are accessible (REF).

Measures

Demographic variables

Age was used as an eligibility criterion; participants aged <18 or >74 were screened out. Participants gave additional sociodemographic information on their gender, education (low: up to 9 years of schooling; medium: at least 10 years (without university entrance qualification); high: at least 10 years (with university entrance qualification), whether they worked in the health sector, and the size of their community (small: < 20,000 inhabitants, medium: 20,001 – 100.000 inhabitants, big: >100.001 inhabitants).

Trust

Trust in governmental and health institutions and trust in the media was assessed ("How much do you trust the following organizations that they are capable of handling the novel coronavirus well and correctly?"; 7-point scale ranging from 1 = "very little trust", 7 = "a great deal of trust"). Trust in health and governmental institutions was averaged across seven institutions (e.g., federal ministry of health, hospitals, local health agencies; Cronbach's alpha = 0.92). Participants could also indicate when they could not make a statement.

COVID-19 related knowledge

We assessed participants' knowledge about SARS-CoV-2 with 3 items about the length of the incubation period (at that time assumed correct: about 14 days), ways of transmission (correct: from human to human), and about pharmaceutical possibilities to treat or prevent it (correct: no medication and no vaccine). Mean knowledge indicates the percentage of correct answers, false and "don't know" answers were coded as wrong answers.

Knowledge about protective behaviours

For several behaviours it was assessed whether participants thought it could prevent COVID-19 infections, such as washing hands for 20 seconds, Not touching the face, using sanitizer, avoiding close contact to infected persons and others. The participants answered questions about the effectiveness of several measures (recoded into 1 = "right", 0 = "wrong"). Since the recommendation at that time was against mask wearing, stating mask wearing is an effective measure was coded as wrong.

Cognitive risk perception

Susceptibility of coronavirus infection was assessed with the following item: "How susceptible do you consider yourself to an infection with the novel coronavirus?", 7-point scale with 1 = "not susceptible at all" to 7 = "extremely susceptible"[1]. Susceptibility correlated considerably both with perceived probability ($r = 0.47$, $p < .001$) and severity ($r = 0.6$, $p < .001$). Thus, these variables were not reported, and susceptibility served as an indicator of cognitive risk perception.

Affective risk perception

Participants evaluated the outbreak situation on a range of affective measures on a 7-point semantic differential: "not scary" to "scary", "not worrying" to "worrying", "rarely thinking on it" to "thinking on it a lot")[2], which were averaged into one variable (Cronbach's alpha = 0.83).

Disease perceptions

Participants evaluated the disease on two 7-point semantic differentials: "slowly spreading - fast spreading" and "close - far away". The second item was reversed for the analysis.

Self-efficacy
Self-efficacy was assessed with one item ("For me, in the current situation avoiding an infection with the novel Coronavirus is.", 7-point scale with 1 = "extremely difficult" to 7 = "extremely easy").

Perception of the outbreak as a media-hype
Participants indicated their perception of the corona outbreak as a media-hype on a 7-point semantic differential ranging from "medially inflated" to "medially insufficiently considered". For the analyses the item was reverse coded with higher values indicating higher perception of the Coronavirus as media-hype.

Protective and crisis-related behaviours
For several behaviours assumed to prevent COVID-19, participants indicated whether they showed the behaviours such as handwashing and covering moth when coughing (1 = "yes", 0 = "no" and "don't know/not applicable").[3] Crises-related behaviours such as buying large quantities of food supplies or cancelling planned travels were assessed the same way.

Acceptance of measures
Acceptance of restriction of freedom served as a proxy for the acceptance of measures ("The government should restrict personal liberties to fight the novel coronavirus."; 7-point scale, ranging from 1 = "strongly disagree" to 7 = "strongly agree").

Time
We included time as a between-subjects variable to assess whether the dependent variables changed over time.

Google trends search volume
Data on google search volume were downloaded from https://trends.google.com (April, 2020). The data represent search interest regarding the term "corona" relative to the maximum interest for the given region (Germany) and time (25/02/2020-24/03/2020). A value of 100 is the peak popularity for the term. A value of 50 means that the term was half as popular. A score of 0 means there was not enough data for this term.

Newspaper volume data
To study the amount of newspaper reporting in Germany during the study period (25.02.2020-24.03.2020) we collected data using the Wiso-Press-Databank with the Search string "Presse Deutschland" to exclude articles from Switzerland and Austria. 158 newspapers, magazines, and online news portals generated hits within the search strings for all texts containing "corona" or "covid". This rough estimate of corona article output in Germany did not exclude double mentioning, which may be due to different editions of local newspapers. However, the data provides an estimate about the presence of the topic in the media.

Participants

Non-probability quota samples were used, representative for the German population regarding age x gender and federal state according to the German census. Participants were members of an ISO 26362:2009-compliant online panel (respondi.de, https://www.iso.org/standard/43521.html). They were compensated for participation by the data collection company at their usual rate.
All individuals between 18 and 74 years of age completing the survey were eligible for inclusion into the analyses. Based on the quotas, participants were admitted to the survey or screened out on the first page. 5,000, 4,781, 6,727, 5,725 participants were contacted by the agency, 973, 966, 1016, 957 participants finished the questionnaire, respectively. Reasons for dropout are unknown. Response rates, defined as the number of those who participated in relation to those having been invited, ranged between 20.2% (wave 2) and 15.1% (wave 3). One participant from wave 3 and wave 4, respectively, was excluded due to previous participation, resulting in n_1=973, n_2=966, n_3=1,015, n_4=956, and N = 3,910 participants. For detailed information on the participants, see supplementary table 1.

Supplementary results

Predicting acceptance of measures – assumptions of linear regressions

Assumption 1: Specificity - Linearity
The rainbow-test tests the null hypothesis, that the relationship between the variables is linear. It is assumed, that this is rather the case in the middle of the distribution. Therefore, a subsample from the middle of the distribution is taken. The rainbow test is rejected ($p<.05$), if the model fit for the whole sample is worse than the fit for the subsample. With reference to supplementary table 2, there is no evidence, that the assumption of linearity is violated.

Assumption 2: No endogeneity
Endogeneity means, that the residuals correlate with the predictors. To check this assumption, we look at the scatterplots (supplementary figure 1) of residuals and predictors of different models. There is an evident negative correlation between the residuals in the second model for acceptance of restrictions and affect. For the other scatterplots, there are no obvious correlations between the residuals and a predictor.

By computing Spearman's rho correlation coefficient, the only significant correlation detected is the one already found in the scatterplot for the second model on acceptance of restrictions (see supplementary table 3). However, it is very small in size and there is no further evidence for the violation of the assumption.

Assumption 3: Homoscedasticity
If the model is fitted well, the residuals (that means, the difference between true and fitted values) should be randomly distributed. This also implies, that the variance of the residuals is homogeneous in the whole range of values. Supplementary figure 2 depicts that the residuals are a bit lower for higher fitted values.

We look at another approach for testing of homoscedasticity: the Goldfeld-Quandt-Test. It compares the variance of two sub models divided in the middle of the sample. If variances differ, the null hypothesis of homoscedasticity is rejected. Supplementary table 4 displays the results. For means of comparison, we look at another approach for testing for homoscedasticity: the Harrison-McCabe-Test (see supplementary table 5). It also compares variances of two subsamples. Again, there is no evidence of violation of the assumption of homoscedasticity.

Assumption 4: No autocorrelation
The Durbin-Watson-Test tests the null hypothesis of no autocorrelation of the residuals. If the test is rejected, we could assume that there is autocorrelation. But this is not the case in the models tested (see supplementary table 6). Thus, the assumption is satisfied.

Assumption 5: Residuals are normally distributed
As residuals are supposed to be random, they should be approximately normally distributed. This assumption can be examined graphically. At first, we look at the histograms for the residuals of different models (supplementary figure 3).
The distribution of the residuals of the first model for affect seems slightly skewed and bi-modal. The residual distribution for the models of acceptance are rather broad-topped. However, the distributions are more or less symmetrical around 0. As this graphical impression is not clear, we additionally look at the quantile-quantile-plots (supplementary figure 4). The red line corresponds to the normal distribution, that means, the actual quantiles follow the theoretical quantiles of the normal distribution quite well. But in case of the models on acceptance of restrictions, the quantiles deviate clearly at the tails.

Assumption 6: No perfect multicollinearity
Multicollinearity is present, if model predictors are correlated. In this case, their variances overlap, and this leads to redundancies in the data and to a loss of information. Supplementary table 7 the pairwise correlations of variables with considerable correlation.

Affect, affective spread, and affective distance are highly correlated. The two variables for trust, as well as the two variables for knowledge are highly correlated, too (see supplementary table 8). It could be considered to reduce the model by using only one of the variables for each concept.

A measure for multicollinearity are variance inflation indicators (VIFs). VIFs greater than 10 are considered problematic. Considerable variance inflation can only be detected in the models with interaction terms. All variance inflation indices for single variables are not problematic (see supplementary table 9). Thus, we can conclude, that we can use all variables in the model and still meet the assumption.

Sensitivity of linear regression results

Ordinal logistic regression
Strictly speaking, the dependent variables on a 7-point Likert scale are not metric, but ordinal. Therefore, we will test in the following, whether the consideration of the true scale of the dependent variable in form of an ordinal logistic regression leads to deviant results.
To this means, we compare the coefficients and confidence intervals of the linear and the ordinal logistic regressions. A significant relationship is indicated with a confidence interval not including 0 in the linear regression and not including 1 in the logistic regression. Thus, we can see whether the same conclusions are drawn in the two different modelling approaches. For the three modes see supplementary table 10,11, and 12.

Model 1: Both models indicate that age, working in the health sector, and later waves are positively related with acceptance of measures. The conclusions drawn are the same in both models. There are no relevant differences.

Model 2: Age, working in the medical sector, own perceived susceptibility, time, trust in institutions and media, and affect are all positively correlated with the acceptance of restrictions. Knowledge and self-efficacy have no clear effect. The conclusions are the same for logistic and linear regression.

Model 3: Again, there are no substantive differences between the logistic and the linear model. Thus, the use of the linear model is justified.

Multiple Imputation

Missing data pattern

Only two of the variables used in the regression models have missings (see supplementary figure 5). The graphic displays, that 3731 cases are complete. Thus, less than 5 % of cases are lost by using Listwise Deletion (LD). In 78 cases, both variables are missing, in 96 cases only trust in media and in 5 cases the composite variable for trust.

Imputation and inspection

We have 3910 observations in total. For each missing value in one or both of the variables on trust, five plausible values are imputed. The variance of the imputations will be used when pooling the results later on to take into account the uncertainty in the imputations for unbiased variance estimates.
Supplementary figure 6 shows scatterplots between age and trust for the observed data (blue) and the five imputed datasets (imputed values are red). The distributions of the imputed values show no obvious pattern.

Comparison of the pooled regression (MI) and Listwise Deletion (LD)

For all the models, we have 3731 (around 95.4 %) cases for LD and 3910 cases, if we use MI. In the following, we inspect whether there are considerable differences between MI and LD. Due to the small proportion of data loss, this is not to be assumed.

Supplementary table 7 shows the differences of Model 1 in case of listwise deletion vs. multiple imputation. It can be concluded that the models are the same: the regression analyses are robust against the use of imputation. Supplementary figure 9 shows that the estimates for the intercept and for knowledge deviate slightly in case of MI. For the other estimators, there are no differences visible. Supplementary figure 10 also shows that there almost no differences in Model 3 with regard to the two methods of dealing with missing values. All in all, due to the small amount of missing data and the marginal differences between MI and LD, the imputation of missing data is not necessary.

2.2. Population groups and acceptance – cluster analysis

Supplementary figure 10 depicts the overall distributions of knowledge of health-protective behaviours and the actual use of these behaviours. Supplementary table 13 lists the variables used for the cluster analysis. For clustering, the following variables have been used:

2.2.1.2 Number of clusters and final partition
K-means is a clustering technique that subdivides the data into a set of k groups. The optimal number of clusters, k, can be determined graphically (see supplementary figures 11 and 12).

The average silhouette width is an estimate of the distance between clusters. Supplementary figure 11 provides indication for the consistency of the clustering classification. In case of appropriate clustering, the distances of each object to other objects within the same cluster ought to be larger than the distances to the neighbouring cluster. Silhouette widths can range from -1 to 1 with higher values indicating an appropriate clustering configuration. The best cluster fit is reached with k=2 clusters, a similar fit is given again for k=6 clusters.

Another indication for the consistency of the clustering classification is the total within sum of squares (see supplementary figure 12). We would like to have a parsimonious partitioning with clearly interpretable clusters and at the same time reach a clear reduction in the total within sum of squares. We see that the reduction decreases for k=2, k=6, and k=10 clusters. This is visible in the slight elbows in the curve. Based on this graphical device and for reasons of interpretability, we decide to partition our sample into four clusters.

Supplementary figure 13A A depicts the four resulting clusters according to the two dimensions considered, namely, knowledge (x-axis) and behaviour (y-axis). Panel B of the same figure depicts the share of participants in each cluster across all four data collections. Supplementary table 14 summarises cluster characteristics.

Supplementary tables

Supplementary table 1. Participants' characteristics across the time points.

	March 03/04 (N=973)	March 10/11 (N=966)	March 17/18 (N=1015)	March 24/25 (N=956)	Total (N=3910)
**Age**					
Mean (SD)	46.4 (15.6)	46.5 (15.7)	46.3 (15.7)	46.0 (16.0)	46.3 (15.7)
Median [Min, Max]	47.0 [18.0, 74.0]	47.0 [18.0, 74.0]	47.0 [18.0, 74.0]	45.5 [18.0, 74.0]	47.0 [18.0, 74.0]
**Gender**					
male	491 (50.5%)	461 (47.7%)	506 (49.9%)	495 (51.8%)	1953 (49.9%)
female	482 (49.5%)	505 (52.3%)	509 (50.1%)	461 (48.2%)	1957 (50.1%)
**Education**					
9 years	106 (10.9%)	102 (10.6%)	121 (11.9%)	96 (10.0%)	425 (10.9%)
10 years (without A level)	350 (36.0%)	336 (34.8%)	375 (36.9%)	325 (34.0%)	1386 (35.4%)
Mind. 10 Jahre (with A level)	517 (53.1%)	528 (54.7%)	519 (51.1%)	535 (56.0%)	2099 (53.7%)
**Community size**					
Small town	365 (37.5%)	366 (37.9%)	408 (40.2%)	370 (38.7%)	1509 (38.6%)
Medium-sized town	256 (26.3%)	244 (25.3%)	250 (24.6%)	234 (24.5%)	984 (25.2%)
Large city	352 (36.2%)	356 (36.9%)	357 (35.2%)	352 (36.8%)	1417 (36.2%)
**Occupation in the health sector**					
no	892 (91.7%)	874 (90.5%)	932 (91.8%)	880 (92.1%)	3578 (91.5%)
yes	81 (8.3%)	92 (9.5%)	83 (8.2%)	76 (7.9%)	332 (8.5%)
**Knowledge on possible treatments**					
wrong	162 (16.6%)	114 (11.8%)	74 (7.3%)	68 (7.1%)	418 (10.7%)
correct	811 (83.4%)	852 (88.2%)	941 (92.7%)	888 (92.9%)	3492 (89.3%)
**Knowledge on transmission**					
wrong	28 (2.9%)	29 (3.0%)	19 (1.9%)	36 (3.8%)	112 (2.9%)

	March 03/04 (N=973)	March 10/11 (N=966)	March 17/18 (N=1015)	March 24/25 (N=956)	Total (N=3910)
correct	945 (97.1%)	937 (97.0%)	996 (98.1%)	920 (96.2%)	3798 (97.1%)
**Knowledge on incubation period**					
wrong	226 (23.2%)	196 (20.3%)	214 (21.1%)	215 (22.5%)	851 (21.8%)
correct	747 (76.8%)	770 (79.7%)	801 (78.9%)	741 (77.5%)	3059 (78.2%)

Supplementary table 2. Results from the Rainbow test.

Model	Rainstatistic	p
Model 1	0.99	0.63
Model 2	1.03	0.27
Model 3	1.02	0.37

Supplementary table 3. Correlation table of selected predictors of the models with residuals.

Model	Predictor	Spearman's rho	p
Model 1	Age	0.02	0.32
Model 2	Trust in institutions	0.04	0.02
Model 3	Knowledge	0.02	0.23
Model 1	Age	0.02	0.32
Model 2	Affective component of risk	0.04	0.02
Model 3	Cognitive component of risk	0.02	0.22

Supplementary table 4. Results of the Goldfeld-Quandt-Tests

Model	GQ statistic	p
Model 1	0.91	0.98
Model 2	0.90	0.99
Model 3	0.90	0.99

Supplementary table 5. Results of the Harrison-McCabe-Tests

Model	HMC statistic	p
Model 1	0.52	0.98
Model 2	0.52	0.99
Model 3	0.52	0.99

Supplementary table 6. Results of the Durbin-Watson-Tests

Model	DW statistic	p
Model 1	2.03	0.81
Model 2	2.03	0.79
Model 3	2.03	0.80

Supplementary table 7. Correlation table between affective component of risk and perceived distance and spreading of the disease.

Measure	Perceived spreading	Perceived closeness	Affective component of risk
Perceived spreading	1.00	0.36	0.32
Perceived closeness	0.36	1.00	0.50
Affective component of risk	0.32	0.50	1.00

Supplementary table 8. Correlation table between trust in institutions and media and knowledge regarding the disease and effective preventive behaviours.

	Trust in media	Trust in institutions	Knowledge regarding protective behaviors	COVID-19 related knowledge
Trust in media	1.00	0.45	0.08	-0.01
Trust in institutions	0.45	1.00	0.10	0.08
Knowledge regarding protective behaviors	0.085	0.10	1.00	0.50
COVID-19 related knowledge	-0.01	0.08	0.50	1.00

Supplementary table 9. Variance inflation Model 3

	**x**
Age	1.09
Occupation in the health sector	1.02
Cognitive component of risk	6.58
Trust in institutions	7.62
Trust in media	7.81
Affective component of risk	1.40
COVID-19 related knowledge	5.55
Self-efficacy	1.20
Time	37.03
Trust in institutions * Time	27.79
Trust in media:* Time	15.43
COVID-19 related knowledge * Time	24.03
Cognitive component of risk	14.89

Supplementary table 10. Model 1 - Ordinal Logistic vs. Linear Model

	OR	2.5 %	97.5 %	beta	2.5 %	97.5 %
Age	1.02	1.01	1.02	0.02	0.01	0.02
Occupation in the health sector	1.19	0.97	1.45	0.18	-0.034	0.40
Time	1.59	1.51	1.68	0.50	0.44	0.55

Supplementary table 11. Model 2 - Ordinal Logistic vs. Linear Model

	OR	2.5 %	97.5 %	beta	2.5 %	97.5 %
Age	1.01	1.01	1.01	0.01	0.01	0.01
Occupation in the health sector	1.29	1.05	1.57	0.27	0.06	0.48
Time	1.39	1.31	1.46	0.34	0.28	0.39
Cognitive component of risk	1.10	1.05	1.15	0.10	0.05	0.14
Trust in institutions	1.14	1.08	1.20	0.10	0.05	0.15
Trust in media	1.14	1.09	1.19	0.13	0.09	0.17
Affective component of risk	1.43	1.36	1.50	0.33	0.28	0.38
Knowledge regarding protective behaviors	0.77	0.54	1.08	-0.27	-0.63	0.10
Self-efficacy	1.03	0.98	1.08	0.03	-0.01	0.08

Supplementary table 12. Model 3 - Ordinal Logistic vs. Linear Model

	OR	2.5 %	97.5 %	beta	2.5 %	97.5 %
Age	1.01	1.01	1.01	0.01	0.01	0.01
Occupation in the health sector	1.28	1.05	1.57	0.27	0.06	0.47
Cognitive component of risk	1.16	1.04	1.29	0.17	0.06	0.27
Trust in institutions	0.84	0.74	0.95	-0.15	-0.27	-0.03
Trust in media	1.38	1.24	1.53	0.30	0.20	0.40
Affective component of risk	1.39	1.33	1.47	0.31	0.26	0.36
COVID-19 related knowledge	0.51	0.27	0.98	-0.78	-1.45	-0.12
Self-efficacy	1.03	0.98	1.08	0.03	-0.01	0.08
Time	0.87	0.63	1.19	-0.09	-0.40	0.23
Trust in institutions * Time	1.13	1.08	1.18	0.10	0.06	0.15
Trust in media * Time	0.93	0.89	0.96	-0.07	-0.10	-0.03
COVID-19 related knowledge * Time	1.27	1.00	1.61	0.28	0.03	0.52
Cognitive component of risk * Time	0.98	0.94	1.02	-0.03	-0.06	0.01

Supplementary table 13. Variables used in the cluster analysis

Correctness of knowledge items	Use of effective behaviors
* Not touching face	* Not touching face
* Use sanitizer	* Use sanitizer
* Stay home when sick	* Stay home when sick
* Cover mouth when coughing	* Cover mouth when coughing
* No close contact to infected persons	* No close contact to infected persons
* Wear face mask	* Wear face mask
* Treatment of covid19	* Buy food in large amounts
* Incubation period of covid19	* buy everyday things in large amounts
* cancel travels	

Supplementary table 14. Cluster characteristics

Cluster statistics	Informed compliant	Complacement	Non-compliant	Ignorant but compliant
Size	855	1157	839	1059
Avg. sil. width	0.16	0.09	0.02	0.18
Avg. dist. within cluster	1.69	1.8	2.26	1.62

Supplementary figures

Supplementary Figure 1. Scatterplot displaying the relationship between residuals and affective (A) and cognitive (B) risks

A

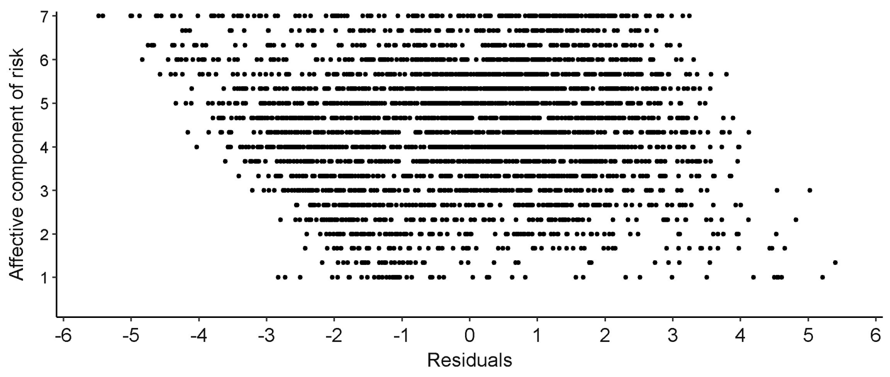

B

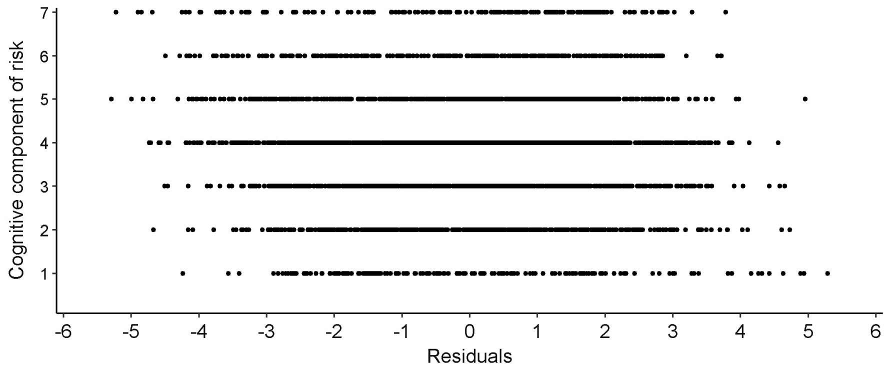

Supplementary Figure 2. Scatterplot displaying the relationship between residuals and predicted values in Model 1 (A), Model 2 (B), and Model 3 (C)

A

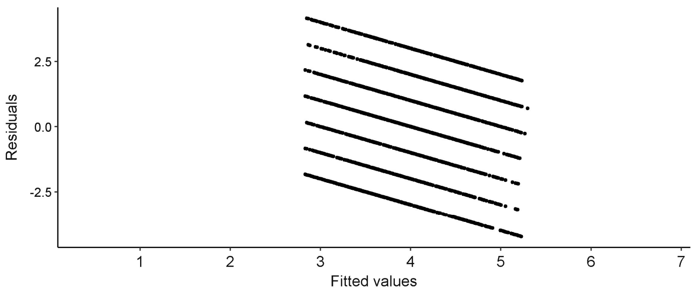

B

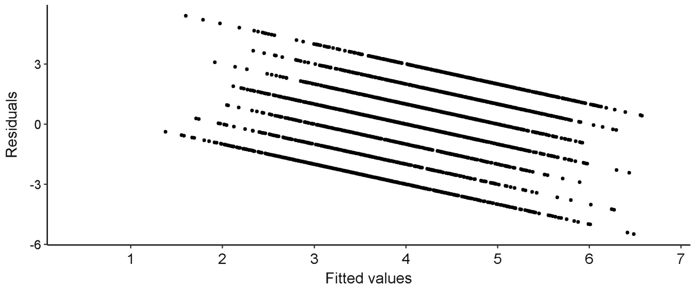

C

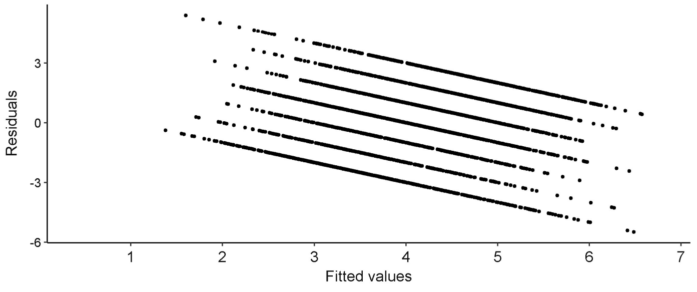

Supplementary Figure 3. Histograms displaying the distribution of residuals in Model 1 (A), Model 2 (B), and Model 3 (C)

A

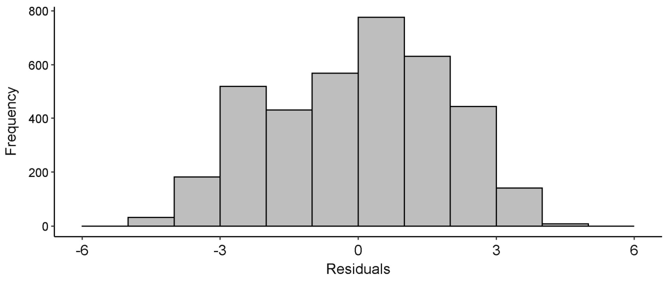

B

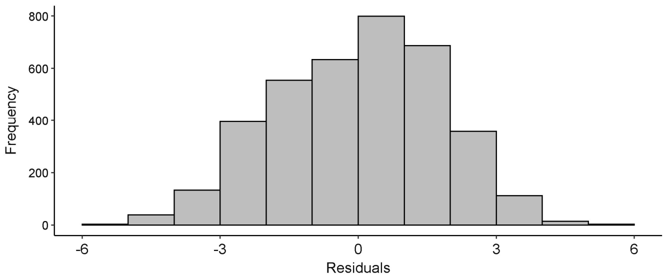

C

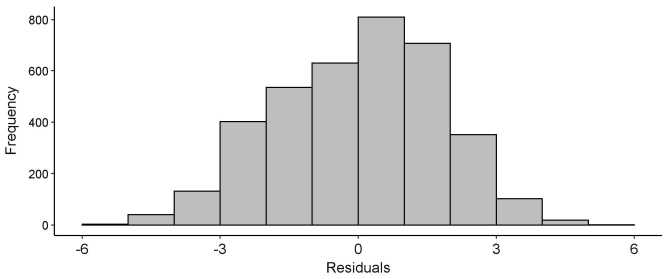

Supplementary Figure 4. Quantile-quantile-plots displaying theoretical and sample quantiles in Model 1 (A), Model 2 (B), and Model 3 (C)

A

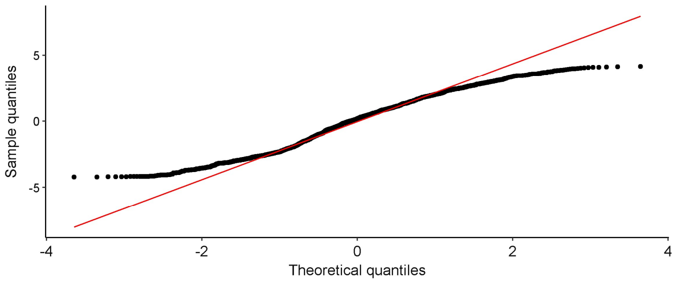

B

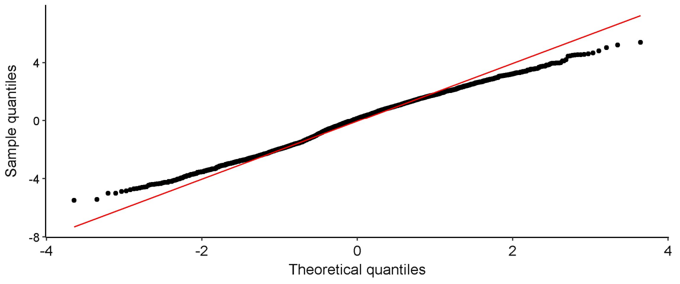

C

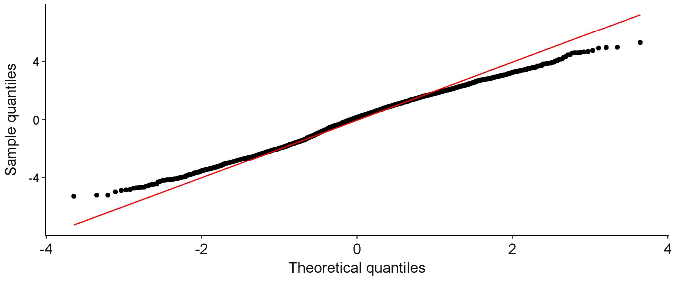

Supplementary Figure 5. Visualising the intersections of missing values in the data set

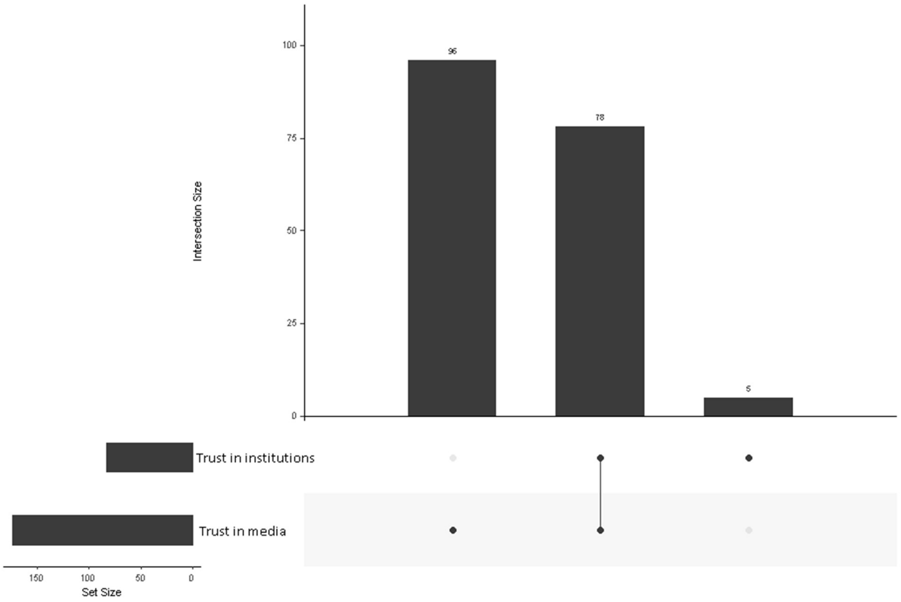

Supplementary Figure 6. Visualising the pattern of missing values in the data set

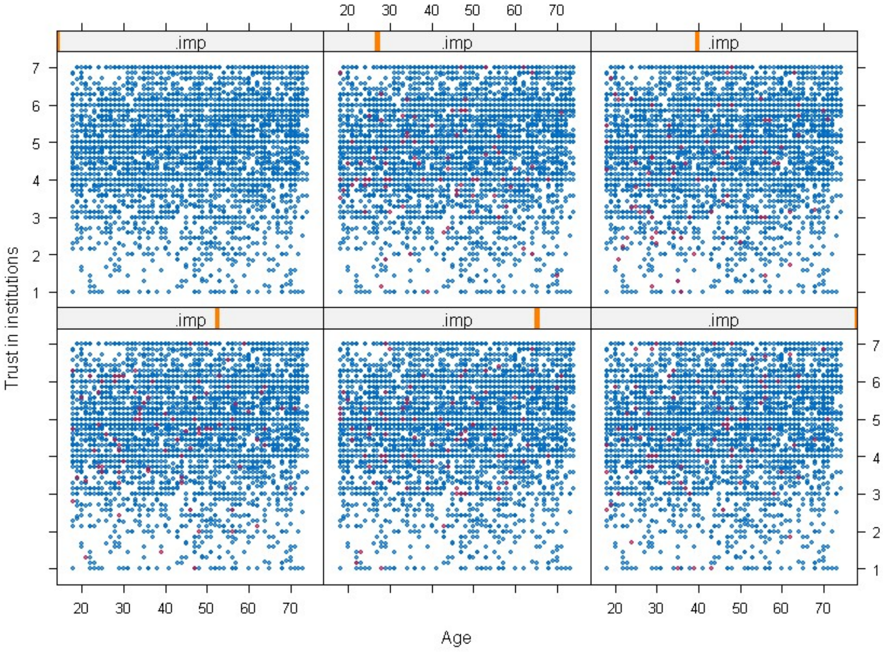

Note. Red dots represent imputed values.

Supplementary Figure 7. Visualising the differences in the estimates (A) and standard errors (B) in Model 1 when deleting missing values vs imputing them

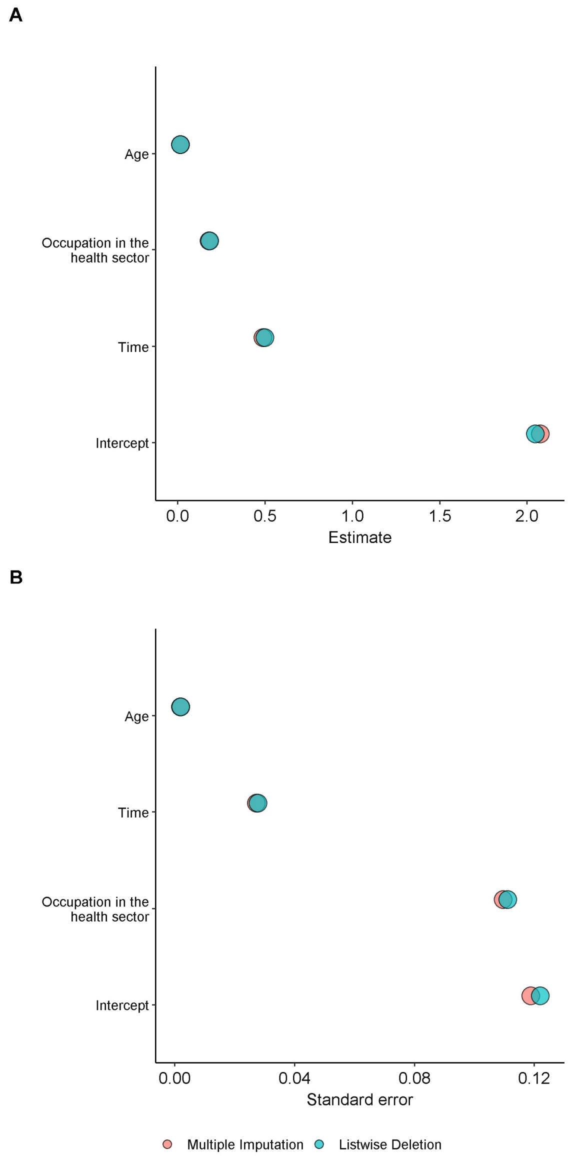

Supplementary Figure 8. Visualising the differences in the estimates (A) and standard errors (B) in Model 2 when deleting missing values vs imputing them

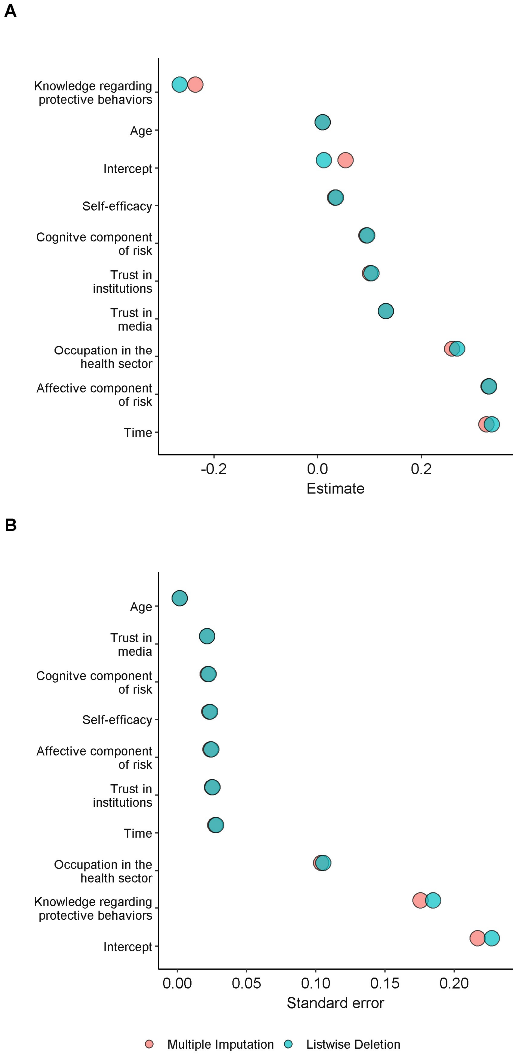

Supplementary Figure 9. Visualising the differences in the estimates (A) and standard errors (B) in Model 3 when deleting missing values vs imputing them

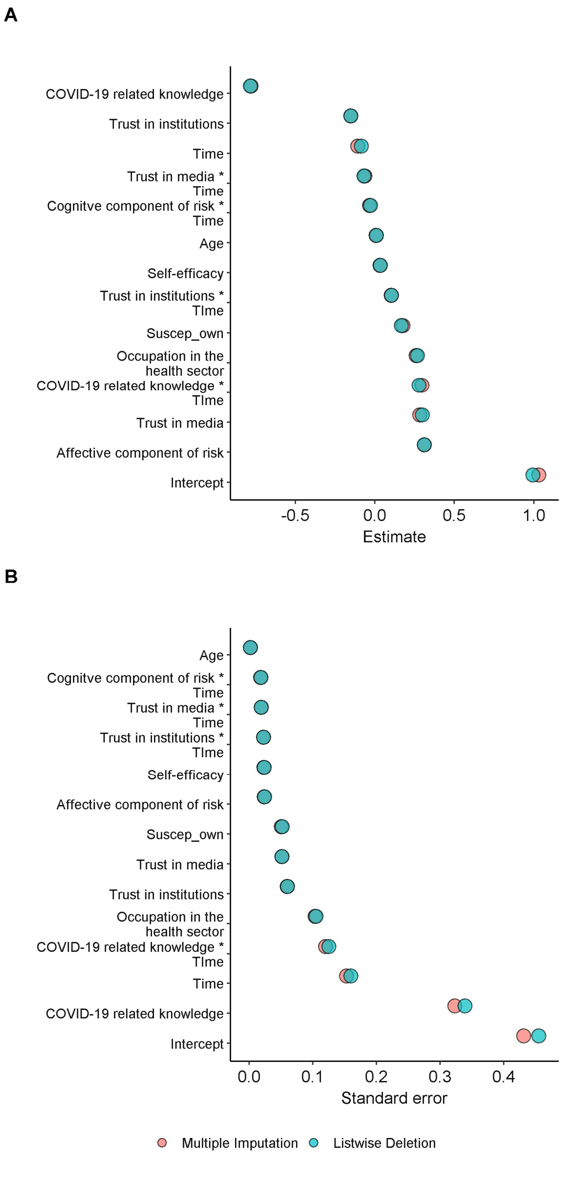

Supplementary Figure 10. Visualising the distribution of (A and B) and the relationship between (C) knowledge regarding protective behaviours and showing protection behaviours.

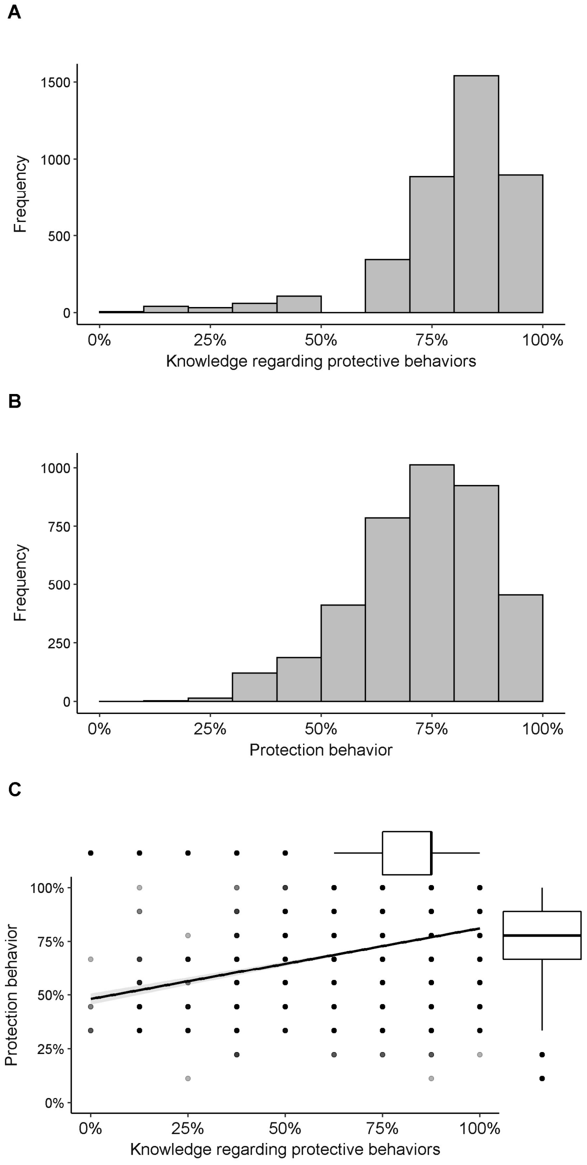

Supplementary Figure 11. Visualising the optimal numbers of clusters k based on the silhouette method.

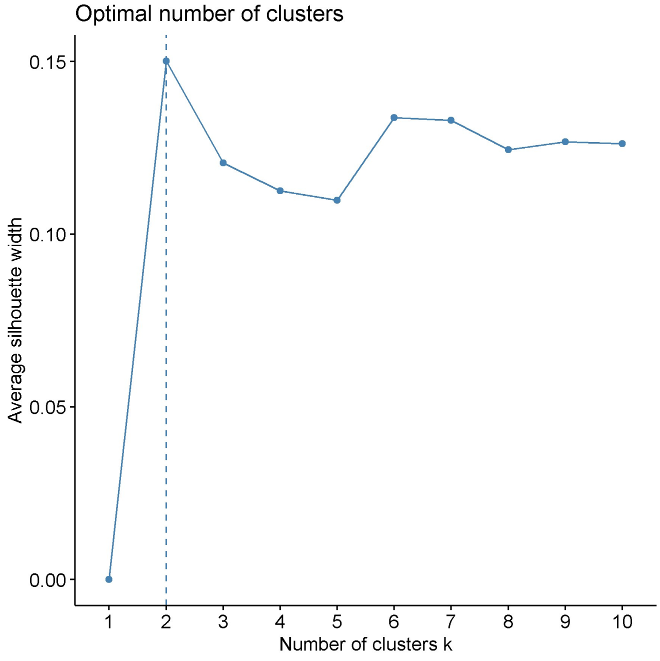

Supplementary Figure 12. Visualising the optimal numbers of clusters k based on the total within sum of squares.

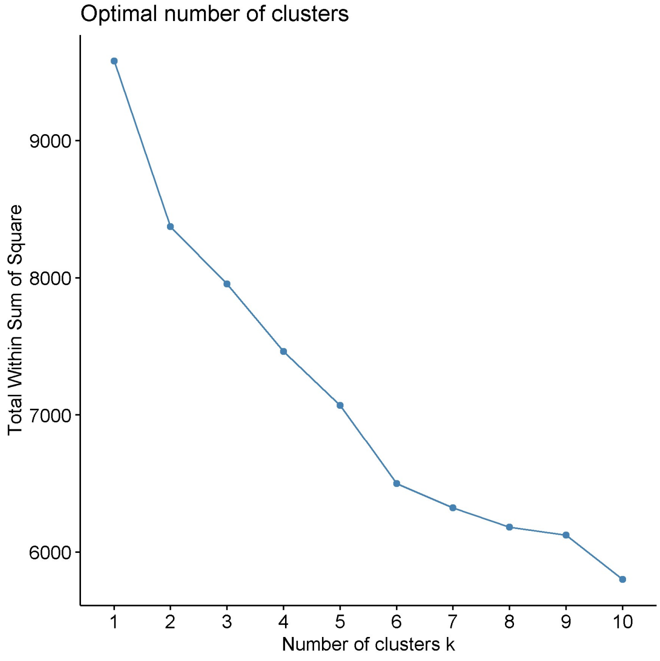

Supplementary Figure 12. Clusters resulting from differences in knowledge about protective measures and actual behaviour and their variation in size over time.

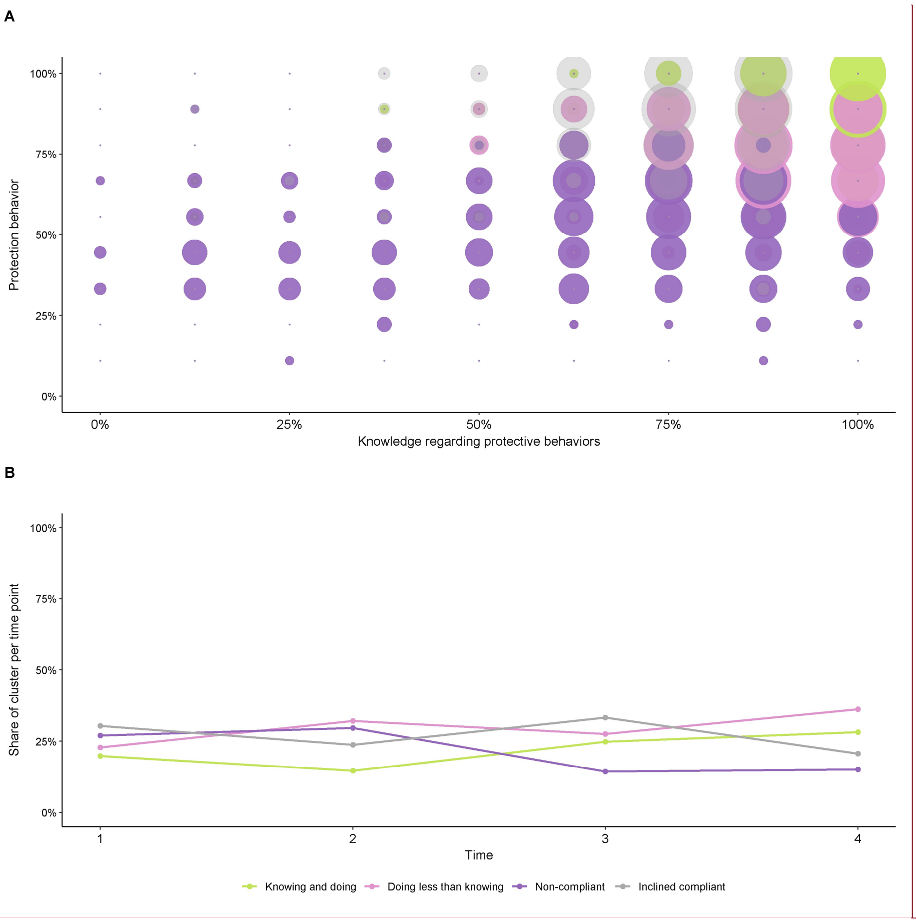

Note. Four clusters resulted from the analysis (left) across all four data collection rounds: (1) informed compliant (knowing and doing); (2) complacent (doing less than knowing); (3) non-compliant (regardless of knowledge); and (4) ignorant but compliant. Compared with all other clusters, the informed-compliant cluster remains relatively small over time. While clusters with high knowledge levels comprise 60% of the sample in Week 4, half the sample still is either not compliant at all or complies less than their knowledge would suggest.

Commented [CB1]: Hier sind noch die alten cluster namen drin

Supplementary references

1 Brewer NT, Chapman GB, Gibbons FX, Gerrard M, McCaul KD, Weinstein ND. Meta-analysis of the relationship between risk perception and health behavior: The example of vaccination. Health Psychology 2007; 26: 136–45.

2 Bradley MM, Lang PJ. Measuring emotion: The self-assessment manikin and the semantic differential. Journal of Behavior Therapy and Experimental Psychiatry 1994; 25: 49–59.

3 Liao Q, Cowling B, Lam WT, Ng MW, Fielding R. Situational Awareness and Health Protective Responses to Pandemic Influenza A (H1N1) in Hong Kong: A Cross-Sectional Study. PLoS ONE 2010; 5: e13350.
